# Cytotoxicity and Antibacterial Efficacy of Betaine- and Choline-Substituted Polymers

**DOI:** 10.1021/acsapm.3c00691

**Published:** 2023-06-13

**Authors:** Lucija Jurko, Damjan Makuc, Alja Štern, Janez Plavec, Bojana Žegura, Perica Bošković, Rupert Kargl

**Affiliations:** ¶ Institute for Chemistry and Technology of Biobased System, Graz University of Technology, Stremayrgasse 9, 8010 Graz, Austria; † Laboratory for Characterization and Processing of Polymers (LCPP), Faculty of Mechanical Engineering, University of Maribor, Smetanova Ulica 17, SI-2000 Maribor, Slovenia; ‡ Slovenian NMR Centre, National Institute of Chemistry, Hajdrihova 19, SI-1000 Ljubljana, Slovenia; § Department of Genetic Toxicology and Cancer Biology, National Institute of Biology, Večna Pot 111, 1000 Ljubljana, Slovenia; ∥ EN-FIST Centre of Excellence, Trg Osvobodilne Fronte 13, 1000 Ljubljana, Slovenia; ⊥ Faculty of Chemistry and Chemical Technology, University of Ljubljana, Večna Pot 113, 1000 Ljubljana, Slovenia; # Department of Chemistry, Faculty of Science, University of Split, Rud̵era Boškovića 33, 21000 Split, Croatia

**Keywords:** hydroxyethyl cellulose, polyvinyl alcohol, antimicrobial, *S. aureus*, *P. aeruginosa*, L929 mouse fibroblasts, cationic polymer

## Abstract

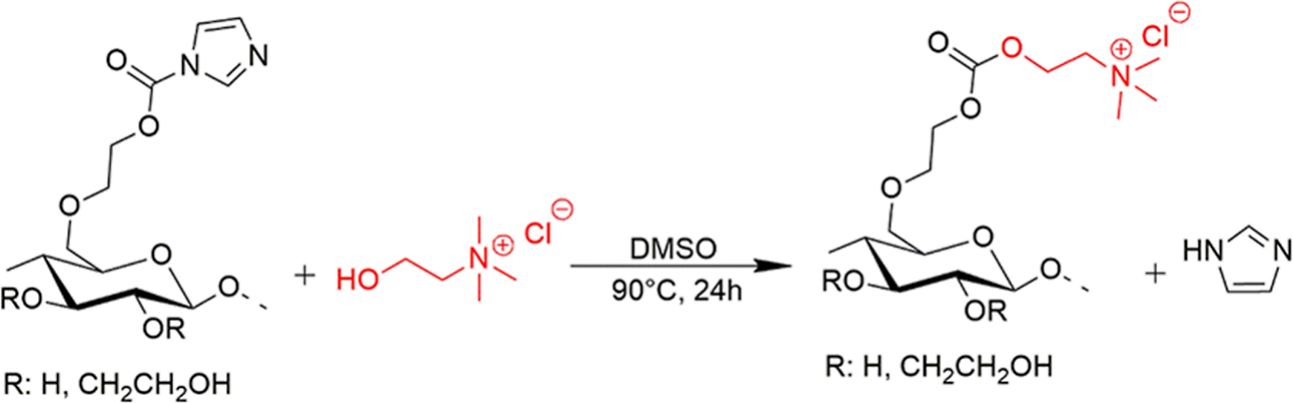

Cationic charge has been widely used to increase polymer adsorption and flocculation of dispersions or to provide antimicrobial activity. In this work, cationization of hydroxyethyl cellulose (HEC) and polyvinyl alcohol (PVA) was achieved by covalently coupling betaine hydrochloride and choline chloride to the polymer backbones through carbonyl diimidazole (CDI) activation. Two approaches for activation were investigated. CDI in excess was used to activate the polymers’ hydroxyls followed by carbonate formation with choline chloride, or CDI was used to activate betaine hydrochloride, followed by ester formation with the polymers’ hydroxyls. The first approach led to a more significant cross-linking of PVA, but not of HEC, and the second approach successfully formed ester bonds. Cationic, nitrogen-bearing materials with varying degrees of substitution were obtained in moderate to high yields. These materials were analyzed by Fourier transform infrared spectroscopy, nuclear magnetic resonance, polyelectrolyte titration, and kaolin flocculation. Their dose-dependent effect on the growth of *Staphylococcus aureus* and *Pseudomonas aeruginosa*, and L929 mouse fibroblasts, was investigated. Significant differences were found between the choline- and betaine-containing polymers, and especially, the choline carbonate esters of HEC strongly inhibited the growth of *S. aureus* in vitro but were also cytotoxic to fibroblasts. Fibroblast cytotoxicity was also observed for betaine esters of PVA but not for those of HEC. The materials could potentially be used as antimicrobial agents for instance by coating surfaces, but more investigations into the interaction between cells and polysaccharides are necessary to clarify why and how bacterial and human cells are inhibited or killed by these derivatives, especially those containing choline.

## 1 Introduction

Cationic polymers are ubiquitous and extensively used in cosmetics (polyquaterniums) and other industries^1^ including medical research.^2^ Many cationic polymers based on amines or quaternary ammonium salts (QAS) may exhibit non-selective antimicrobial or other cytotoxic properties.^3−5^ Generally, QAS and especially protonated (poly-) amines are expected to bind ionically to negatively charged cell membranes, causing their destabilization. This may lead to membrane rupture and consequently to cell penetration or lysis.^6^ Such effects might also be desired in the case of targeted drug delivery when cell membranes need to be crossed.^7^ By covalently binding QAS to polysaccharides, antimicrobial materials have been obtained.^8^ The most commonly used compounds for commercial cationization are probably 3-chloro-2-hydroxypropyl trimethylammonium chloride^9^ or glycidyl-3-methyl ammonium bromide or equivalent halides, but these materials are usually not used as antimicrobial agents or might not be effective.^10^ In this work, alternatives to these cationic substituents are investigated.

Many quaternary nitrogen compounds are essential constituents of living organisms, and glycine betaine (or trimethyl glycine) is found in plants, where it is involved in osmoregulation and metabolic processes.^11^ Glycine betaine esters with fatty alcohols are known to be antimicrobials and are susceptible to hydrolysis of the ester bond at neutral to alkaline pH values.^12^ Hydrolytic stability might be the reason why only a few publications reported the esterification of polysaccharides with glycine betaine. Stability might also be the reason that glycine betaine esters, unlike phosphocholine esters, have not been described as components of cell membrane lipids.^13^

Starch esters with betaine were prepared,^14^ whereas cellulose^15,16^ betaine esters were used as flocculants, and hydroxyethyl cellulose^17^ betaine esters served as mucoadhesives. These products were prepared by a two-step reaction with thionyl chloride. Tosyl chloride activation was also described for betaine-modified cellulose powders under more heterogeneous conditions.^18^ Structurally related to betaine hydrochloride is the compound choline chloride, which is an important motif in neurotransmission,^19^ in phospholipids, and in many bacterial polysaccharides.^20^ Choline esters appear to be more hydrolytically stable than those of betaine.^21^ Some publications report the use of choline chloride in polymer modification as part of a deep eutectic solvent^22^ or for polyethylene glycol.^23^

To contribute to a better understanding of the properties of betaine and choline esters, this work describes the esterification of semisynthetic hydroxyethyl cellulose (HEC) and fully synthetic polyvinyl alcohol (PVA) with glycine betaine hydrochloride or choline chloride in polymer analogous reactions. PVA was used for comparison due to its usually higher heat stability and the absence of glycosidic bonds, which could reduce side reactions and simplify purification and analysis. Reactions were carried out in dimethyl sulfoxide using 1,1′-carbonyldiimidazole (CDI) as a coupling agent, avoiding thionyl chloride in the laboratory. ^1^H and ^13^C nuclear magnetic resonance (NMR), infrared spectroscopy, polyelectrolyte titration, flocculation, and elemental analysis were used to elucidate the molecular structure and composition of the polymer derivatives. Their concentration-dependent biocompatibility with mouse L929 fibroblasts and their antimicrobial properties against *Staphylococcus aureus* and *Pseudomonas aeruginosa* were assessed and compared to the unbound substituents and to commercial cationic HEC.

## 2 Materials and Methods

### 2.1 Materials

Hydroxyethyl cellulose (HEC, *M*_W_ = 90,000 g/mol), polyvinyl alcohol (PVA, 89–90% hydrolyzed *M*_W_ = 89,000 g/mol), betaine (BET), betaine hydrochloride (BET HCl), dimethyl sulfoxide (DMSO) anhydrous, 1,1′-carbonyldiimidazole (CDI), sodium chloride (NaCl), sodium hydroxide (NaOH; 0.1 M), hydroxyethylcellulose ethoxylate, cationic HEC (q-HEC; LOT: 525944), toluidine blue (certified), polystyrene sulfonic acid, sodium salt (PSS), sodium pyruvate, l-glutamine, dimethyl sulfoxide (DMSO), and penicillin/streptomycin (1%) were purchased from Sigma-Aldrich (Merck KGaA, Darmstadt, Germany). Choline chloride (Ch-Cl) was purchased from Acros (Geel, Belgium). Acetone was purchased from Carlo Erba (Val-de-Reuil, France). Dialysis tubes (regenerated cellulose membrane, MWCO 14 kDa) were purchased from Carl Roth (Karlsruhe, Germany). Milli-Q water from a Millipore (MA, USA) water purification system (resistivity ≥ 18.2 MΩ cm, pH 6.8) was used for the preparation of all aqueous solutions. Fetal bovine serum (100500-064) and minimum essential medium (MEM; 51200–046) were from Gibco (Amarillo, TX, USA). Trypsin was from Invitrogen (Waltham, MA, USA). Phosphate-buffered saline was from PAA Laboratories (Toronto, Canada). 3-(4,5-Dimethylthiazol-2-yl)-2,5-diphenyltetrazolium bromide (MTT) was from Abcam (Cambridge, United Kingdom).

### 2.2 Esterification of Hydroxyethyl Cellulose and Polyvinyl Alcohol with Betaine Hydrochloride and Choline Chloride

#### 2.2.1 Betaine Esters (b-HEC and b-PVA)

Betaine hydrochloride (BET HCl) (1280 mg, 8.27 mmol) was added to 12 mL of DMSO, then heated to 90 °C, stirred at 200 rpm, and left to wet for 30 min in the form of a dispersion. BET HCl does not dissolve in DMSO under these conditions.

Carbonyldiimidazole (1324 mg, 8.17 mmol) was added to 12 mL of the above solution to activate BET HCl for 30 min. This led to a complete dissolution of the compound formed as proposed in Scheme S1. The formation of gaseous CO_2_ was observed for 30 min. The reaction was prepared twice in 12 mL each.

**Scheme 1:**
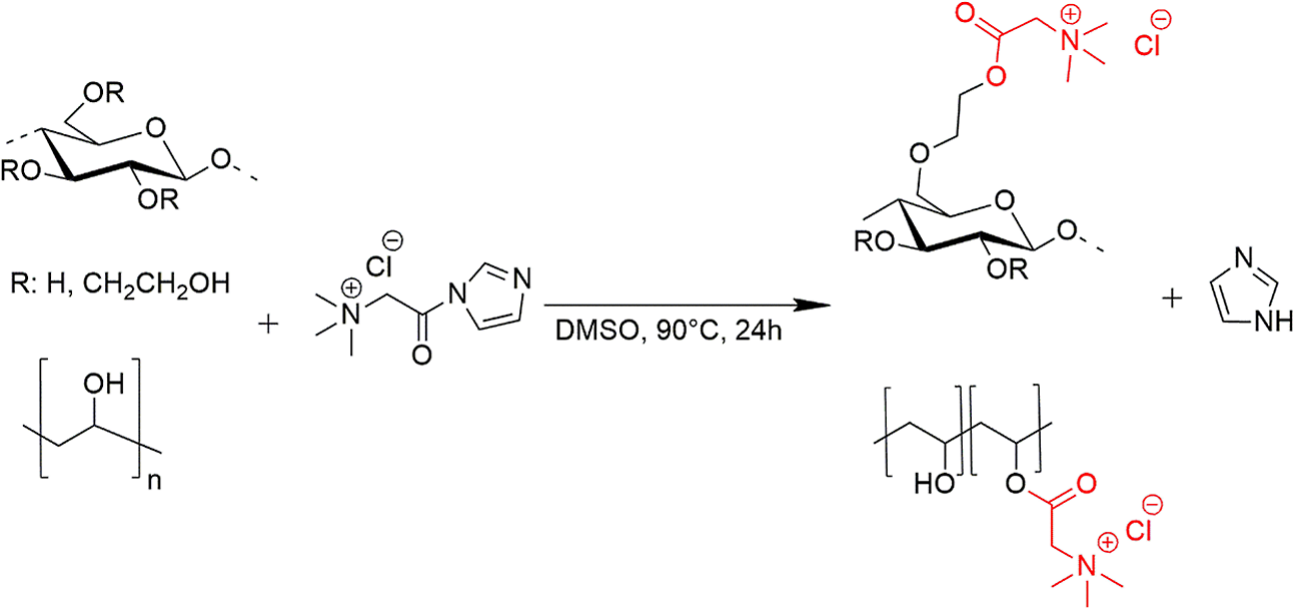
Scheme for the Quaternization of (a) Hydroxyethyl Cellulose and (b) Polyvinyl Alcohol with Betaine Chloride (Red)

HEC (500 mg, 2.44 mmol AGU, assuming a degree of hydroxy ethylation of 3) was dissolved in 12 mL of DMSO at room temperature under stirring at 200 rpm until a clear solution was obtained. PVA (500 mg, 11.35 mmol OH) was dispersed (it does not dissolve at room temperature) in 12 mL of DMSO under stirring at 200 rpm. 12 mL of either HEC solution or PVA dispersion was slowly mixed with 12 mL of the activated BET solution and reacted for 24 h at 90 °C under stirring at 200 rpm (Scheme 1).

All two reaction solutions became clear upon mixing but turned dark brown after approximately 3 h of reaction and remained constant thereafter.

#### 2.2.2 Choline Ester (c-HEC)

**Scheme 2:**
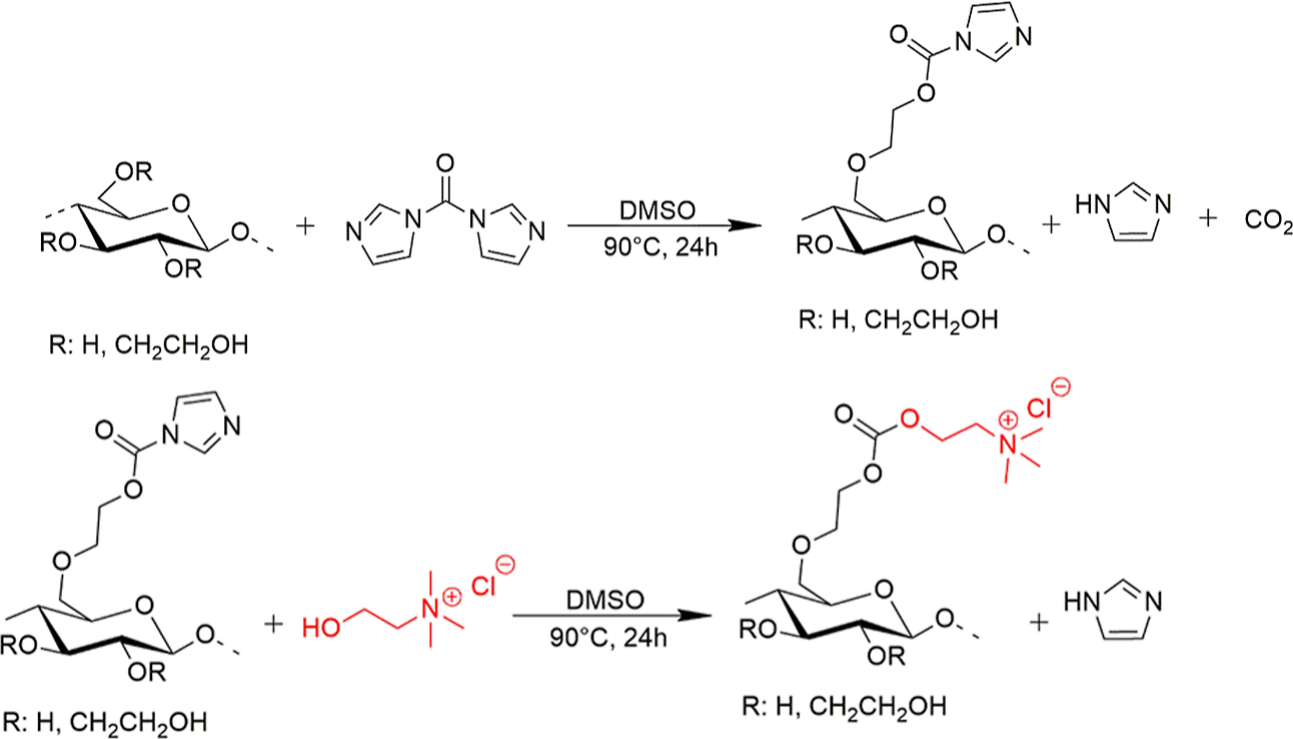
Proposed Reaction of Hydroxyethyl Cellulose (HEC) with Carbonyldiimidazole (CDI) (Above) and Activated HEC with Choline Chloride (CH–Cl) (Below)

Hydroxyethyl cellulose (HEC) (1050 mg, 5.11 mmol AGU, assuming a degree of hydroxy ethylation of 3) was added to 75 mL of DMSO, stirred at 200 rpm, and left to dissolve overnight. Carbonyldiimidazole (2572 mg, 15.86 mmol) was added to the above 75 mL solution and heated at 90 °C with stirring at 200 rpm to activate HEC for 30 min, resulting in a clear solution and the formation of gaseous CO_2_ (Scheme 2). Choline chloride (3.5 g, 25.07 mmol) was added to the reaction solution and allowed to react at 90 °C for 24 h. The reaction of PVA with choline chloride was unsuccessful due to cross-linking of the hydroxyl group of PVA and the resulting insolubility of the obtained material in water. The choline-PVA derivative was therefore not included in this study even though the cross-linked material and the method could still be useful for other purposes such as scaffolds or wound dressings. This cross-linking was not observed for HEC. There are two explanations for the observed differences, first, PVA only contains secondary hydroxyls which could be less reactive toward CDI, leading to a higher number of underivatized free hydroxyls in the first place. These free hydroxyls subsequently react with the acyl-imidazole-activated hydroxyls to form carbonate bonds, resulting in a dense cross-linked structure. Second, compared to HEC, PVA might already become insoluble at a lower cross-linking density due to the more compact and non-polar backbone.

#### 2.2.3 Product Purification

All reaction solutions were cooled to room temperature and poured into 100 mL of acetone, which caused precipitation of the products and likely of unmodified or activated betaine, choline, or polymer but not imidazole. The precipitates were centrifuged (9000 rpm, 30 min), the supernatant was removed, and the raw product was washed with 3 × 50 mL of acetone after each centrifugation step. The final isolates were dried overnight in a vacuum oven (40 °C, 100 mbar). The dried products were re-dissolved in ultrapure water (30 mL), and the pH was adjusted to 7. The aqueous solution was dialyzed (regenerated cellulose membrane, MWCO 14 kDa) for 12 h against 2 M NaCl and 36 h against ultra-pure water to remove unbound small molecules and then lyophilized (−35 °C; ∼2.0 × 10^–3^ mbar, 4 days) to obtain the final powdered light brownish coarse product. The obtained materials are labeled b-HEC, b-PVA, and c-HEC and compared to commercial cationic hydroxyethyl cellulose ethoxylate (q-HEC). The HEC or PVA polymer recovery, i.e., the mass of the polymer backbone in the modified final products, was calculated according to eq 1, to be able to compare yields within products of different degrees of substitution (DS).
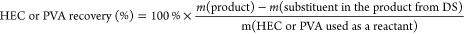
1

### 2.3 Attenuated Total Reflectance Infrared Spectroscopy

Attenuated total infrared reflectance infrared spectroscopy (ATR-IR) spectra of all samples were recorded using a PerkinElmer FTIR System Spectrum GX Series-73565 at a scan range of 4000–650/cm. A total of 32 scans were performed for all measurements with a resolution of 4/cm.

### 2.4 NMR Spectroscopy

NMR measurements were carried out on a Bruker Avance Neo 600 MHz NMR spectrometer equipped with a 5 mm BBO probe. NMR samples were prepared by dissolving ca. 50 mg of the sample in 0.6 mL of D_2_O. NMR chemical shifts are reported in δ (ppm) relative to TMS (δ ∼ 0 ppm). Identification was performed using the characteristic NMR resonances, assigned based on their chemical shifts as well as with the use of a set of the following 2D experiments: heteronuclear single-quantum coherence (^1^H–^13^C HSQC) and heteronuclear multiple bond correlation (^1^H–^13^C HMBC). 1D ^1^H NMR spectra were recorded using the zgesgp standard pulse sequence. The spectral width was set to 11,904 Hz (19.85 ppm). A relaxation delay of 1.0 s was used. ^1^H NMR spectra were recorded using 65,536 points and 16 scans. ^13^C NMR data were acquired using the deptqgpsp pulse sequence. The spectral width was set to 35,714 Hz (236.68 ppm). A relaxation delay of 1.0 s was used. ^13^C DEPTQ spectra were recorded using 32768 points and 30000 scans. 2D ^1^H–^13^C HSQC spectra were acquired using the hsqcedetgpsp pulse sequence. The spectral width for the proton dimension was 7812 Hz (13.0 ppm) and that for the carbon dimension was 30,177 Hz (201 ppm). The HSQC spectra were recorded using 2048 points in F2 and 256 increments in F1 dimension and 16 scans. 2D ^1^H–^13^C HMBC spectra were recorded using the hmbcetgpl3nd pulse sequence. The spectral width for proton dimension was 7812 Hz (13.0 ppm) and that for carbon dimension was 36,216 Hz (241 ppm). The HMBC spectra were recorded using 2048 points in F2 and 400 increments in F1 dimension and 48 scans. A relaxation delay of 1.00 s was used for both 2D experiments. For comparison, a polyvinyl alcohol with a lower molecular weight (PVA Mowiol 8–88, *M*_w_ ∼ 67,000 Da, 10.0–11.6% residual content of acetyl, Sigma-Aldrich) than those used for derivatization was dissolved in D_2_O and measured as a control (Figures S21 and S22).

### 2.5 Polyelectrolyte Charge Titration, Flocculation, Elemental Analysis, and DS

Charges present in the products bound to the backbone were measured by polyelectrolyte titration in water. This titration method usually does not detect charges of small molecules because no large charge complexes are formed with the titrant. It therefore is indicative for the presence of polyelectrolytes. The products were titrated with anionic sodium polystyrene sulfonate (PSS, 1 mmol/L). After incremental addition of the titrant (0.1–0.25 mL), with an autotitration unit DL 53 (Mettler Toledo, Switzerland), the equivalence point was determined with a phototrode DP5 (Mettler Toledo, USA) at a wavelength of 660 nm with addition of toluidine blue as an indicator (*c* = 0.1 mmol/L). The degree of substitution with the quaternary ammonium compound (and therefore the known mass of the substituent) was calculated from the amount of cationic charge obtained from the polyelectrolyte titration and the known residual molar masses of the monomeric unit of the polymers.

The degree of substitution (DS) of betaine or choline chloride was calculated by analyzing the nitrogen content (*N* %) of a sample with a typical mass of 2 mg using the “Elemental Analyzer vario MICRO cube” from Elementar. Calibration was done using sulfanilamide. Helium was the carrier gas. DS was calculated according to eq 2
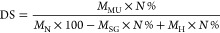
2where *M*_MU_ is the molar mass of one unsubstituted monomeric unit (294.3 g/mol for HEC, 44.05 g/mol for PVA), *M*_N_ is the molar mass of nitrogen (14.01 g/mol), *M*_SG_ is the molar mass of the residual substituent without the oxygen, that is part of the backbone, betaine hydrochloride (136.61 g/mol), choline chloride carbonate (166.63 g/mol), and *M*_H_ is the molar mass of hydrogen and accounts for the fact that the molar mass of the unsubstituted monomeric unit of the backbone is decreased by DS × molar mass of hydrogen upon substitution.

The flocculation capacity was evaluated by preparing a kaolin suspension at a concentration of 0.25 wt % in water and adding different amounts of the samples (0.5–6 mg/mL). The samples were stirred at 200 rpm for 10 min and then at 40 rpm for 5 min. After stirring, the samples were allowed to settle for 5 min. A pH of 6 was measured and remained constant. Transmittance of the supernatants of each solution was measured at 420 nm using a Cary 60 UV–visible Spectrophotometer (Agilent Technologies, California, USA) with paired quartz cuvettes (optical path 1 cm). Solutions with higher transmittance presented higher flocculation performance of the cationic polymers.^24^

### 2.6 Evaluation of Biological Efficacy

#### 2.6.1 Biocompatibility Testing

The biological reactivity and potential cytotoxic activity of HEC, PVA, and their betaine and choline derivatives were evaluated on mouse fibroblasts (NCTC clone 929: CCL 1; L929; American Type Culture Collection, ATCC, Manassas, VA, USA) in vitro in accordance with the International Standard ISO 10993-5:2009, Biological evaluation of medical devices—Part 5: Tests for in vitro cytotoxicity, as previously described.^5^ Biological reactivity was evaluated by light microscopy, following exposure to the samples for 24 ± 1 and 48 ± 1 h, and rated on a scale of 0–4 as described in ISO 10993-5:2009. The samples were dissolved in complete growth medium (MEM supplemented with 10% FBS, 4 mM l-glutamine, 0.11 mg/mL sodium-pyruvate, 100 IU/mL penicillin, and 100 μg/mL streptomycin) at the following concentrations: 20 mg/mL for HEC, b-HEC, q-HEC, BET HCl and Ch-Cl, and 2 mg/mL for c-HEC and b-PVA. Graded concentrations in the range of 0.002–20 mg/mL of HEC and the derivatives of HEC and PVA were tested. PVA was not soluble in the complete growth media, at the tested concentration range, and could therefore not be tested. In each experiment, a negative control (complete growth media) and a positive control (5% DMSO) were included. Three independent experiments were performed in five replicates. The program GraphPad Prism 9 (GraphPad Software, San Diego, CA, USA) was used for statistical analysis and calculation of half-maximal inhibitory concentration (IC_50_) values, as previously described.^5^ Light microscopy images of the cells after exposure to graded concentrations of the tested samples are shown in the Supporting Information (Figures S24–30).

#### 2.6.2 Antimicrobial Testing

All samples were dissolved in sterilized distilled water at a stock concentration of 400 mg/mL except c-HEC (200 mg/mL) and q-HEC (100 mg/mL) due to high viscosity or lower solubility. From these solutions, a two-fold dilution series was prepared. From each concentration of the dilution series, 0.100 mL was added to 1.9 mL of liquid in the test tube containing the microorganism [*S. aureus* (DSM 799) or *P. aeruginosa* (DSM 1128)]. The final concentration of the substances to be tested in contact with the microorganism ranged from 20 to 0.02 mg/mL, except for c-HEC (10–0.01 mg/mL) and q-HEC (5–0.005 mg/mL). The test tubes were incubated at 37 °C for 24 h, and the growth of the bacteria was observed visually and expressed as (−) complete growth inhibition, (+) partial growth inhibition, (++) no growth inhibition, and (*) debris in the tested samples. Two repetitions were performed for each concentration, and the minimum inhibitory concentration (MIC) and minimum bactericidal concentration (MBC) were determined. In the case where growth could not be accurately determined in the liquid media, the polymer solutions were applied onto solid media containing the microorganisms for an approximate determination of MIC, MBC.

## 3 Results and Discussion

### 3.1 Esterification and Characterization with ATR-IR

All esterified products are well water soluble. Purified b-PVA, b-HEC, and c-HEC are light brown powdered products, in comparison to white powdered products of q-HEC.

Figure S1 shows the comparison of all products with the starting materials. A peak at 1740 cm^–1^, characteristic for the carbonyl stretching vibration of esters and carbonates, is visible. A shift of C=O (1740 cm^–1^) compared to C=O of betaine hydrochloride (1715 cm^–1^) and the same peak absent for q-HEC support the hypothesis of ester formation. Hydroxyl moieties (3340 cm^–1^) are observed in all samples but less pronounced for betainates. The strong band at 1190 cm^–1^ in c-HEC can be addressed to the C–O vibration of the carbonates stemming either from the reaction with choline or with residual HEC hydroxyls. In the latter case, intra- and intermolecular cross-links could be formed. Due to the large access of CDI in the activation step of HEC, and the solubility of the final products, it is assumed that cross-linking is sufficiently suppressed. The band at 1049 cm^–1^, associated with C–O stretching vibrations of the anhydro glucose unit, is observed for all HEC samples.^25^ Smaller peaks at 1633 cm^–1^ are observed for b-HEC and b-PVA which correspond to the C=O stretch of deprotonated BET. Compared to the C=O stretch vibration of BET HCl at 1715 cm^–1^, there is a large shift caused by the protonation of the carboxylate into the hydrochloride salt form.

### 3.2 NMR Spectra

#### 3.2.1 HEC Betaine Ester (b-HEC)

The carbonyl carbon signal of b-HEC can be observed at δ 167.6 ppm (Figure S11), slightly upfield compared to BET HCl (δ 170.2 ppm) (Figure S3). There is a downfield shift from δ 3.8 ppm (Figure S6) to δ 4.4 ppm (Figure S10) for the H8 HEC proton, which is due to the deshielding effect of the nearby oxygens of ester groups. The methylene group of covalently bound betaine (peak H10, Figure S10) can be observed at the same value as the CH_2_ group of the HEC peak H8, Figure S10). Comparing the carbon peak at δ 63 ppm of the same methylene group of b-HEC (C8, Figure S11), no chemical shift change is observed for the C8 peak of unmodified HEC (Figure S7). The methylene group of betaine is observed at δ 66.8 ppm (Figure S3).

**Figure 1:**
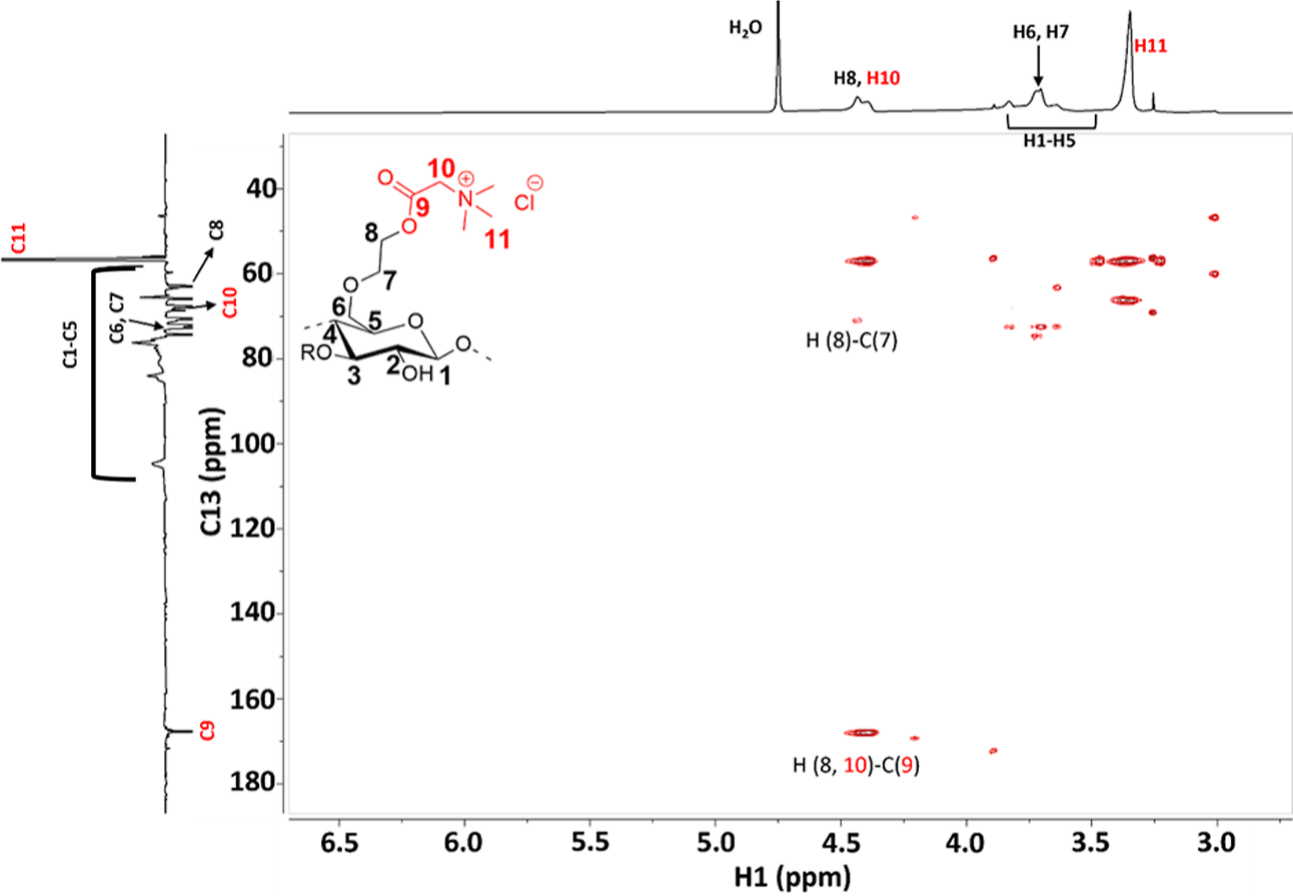
^1^H–^13^C HMBC NMR spectra of b-HEC.

The^1^H–^13^C HSQC spectra of b-HEC (Figure S18) show clear correlations between protons attached to the carbons of CH- groups of HEC or those of betaine. Peaks of the HMBC spectra (Figure 1) give insights into correlations between the protons and carbons of HEC and covalently bound betaine; specifically, H (8)–C (6,7) confirm the hydroxyethyl substituent, and H (8,10)–C (9) correlations confirm a covalent bond between betaine and the polymer.

#### 3.2.2 PVA Betaine Ester (b-PVA)

The CH_2_ groups of unmodified PVA 89–90% hydrolyzed *M*_W_ = 89,000 g/mol (H1 and C1) are identified at δ 1.92 ppm and δ 63 ppm, respectively (Figures S8 and S9). The same H1, H1′ and C1, C1′ signals of modified PVA are observed in the range of δ 1.5–2.2 ppm and δ 44–50 ppm (Figures S12 and S13). Deshielding effects of the substituent cause a downfield shift of peaks, which is attributed to protons of modified PVA from 3.72 ppm (Figure S8) to 5.22 ppm (H2′ signals, Figure S12). Depending on the molecular weight of the polymer, different intensities are seen for PVA, as could be shown by measuring a 67 kDa Mowiol 8–88 PVA (Figure S21). The peak for the carbon (C1) is observed at 48 ppm (Figure S22). Due to the higher molecular weight of the polymer used in the reaction (PVA, 89–90% hydrolyzed *M*_W_ = 89,000 g/mol Figure S9), the intensity of certain signals is lower and probably not visible compared to the lower molecular weight PVA.

The methyl groups of the quaternary nitrogen are seen at δ 3.4 and 57 ppm. Furthermore, ^1^H NMR of b-PVA shows the presence of unreacted betaine, which is supported by NMR signals observed at δ 3.26 and δ 3.90 ppm (Figure S12) and the IR spectra. However, peaks of unreacted betaine could not be identified in the ^13^C NMR spectra (Figure S13) due to overlapping of the signals with b-PVA.

**Figure 2:**
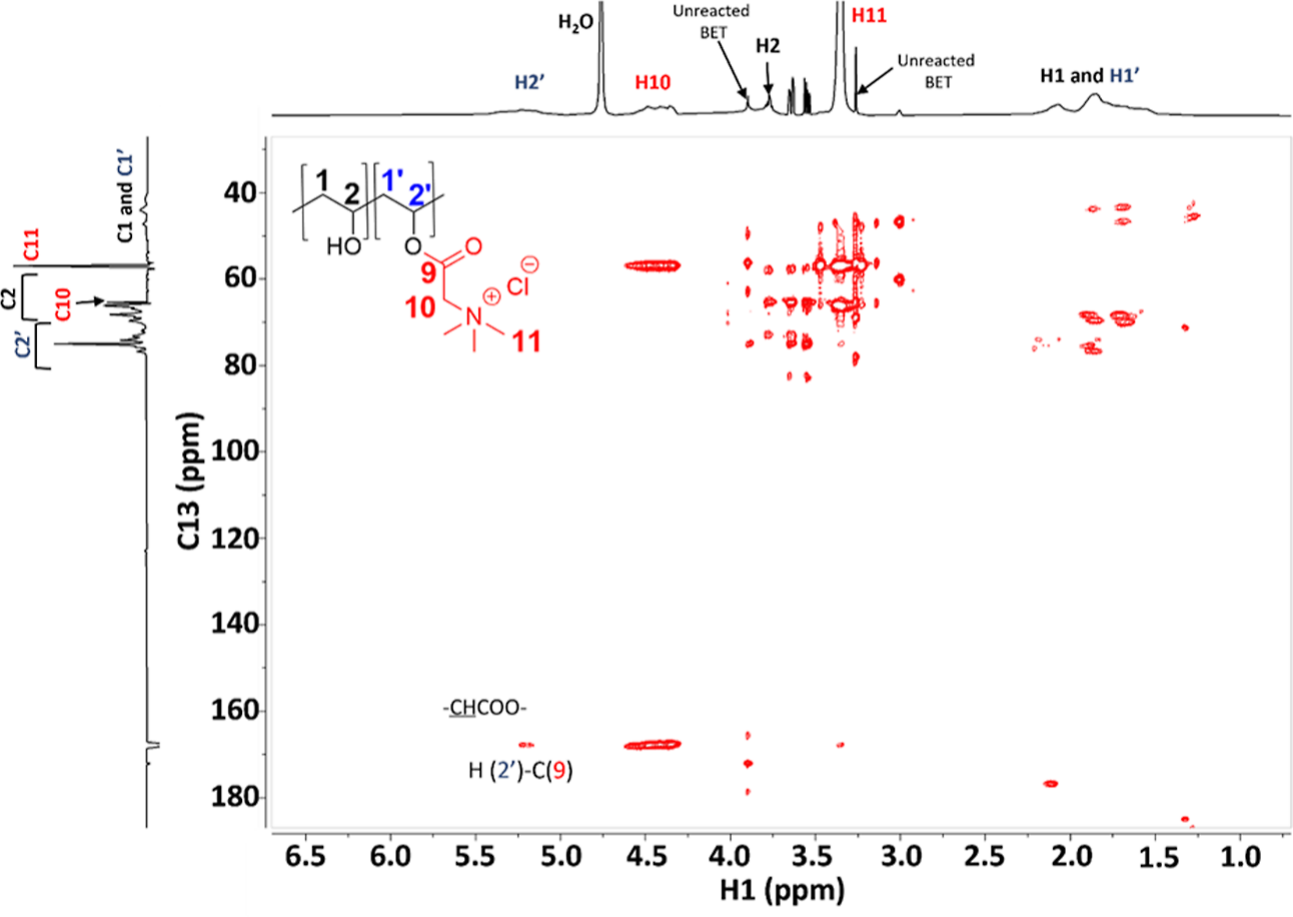
^1^H–^13^C HMBC NMR spectrum of b-PVA.

^1^H–^13^C HSQC spectra of b-PVA (Figure S19) and the ^1^H–^13^C HMBC spectra (Figure 2) show single bond and long-range correlation signals between protons and carbons of their own and neighboring groups of PVA and betainate. A covalent bond between PVA and betaine was confirmed by the correlation between H (2′)–C (9).

#### 3.2.3 HEC Choline Ester (c-HEC)

A covalently bound choline can be assumed with the carbon of the carbonate ester group observed at δ 154.6 ppm (Figure S15). CH-groups of the cellulose backbone are spread over a wide region at δ_C_ 76–77, δ 84, and δ 105 ppm in the ^13^C NMR spectrum (Figure S15), as well as between δ 3.6 and δ 3.9 ppm in the ^1^H NMR spectrum (Figure S14).

**Figure 3:**
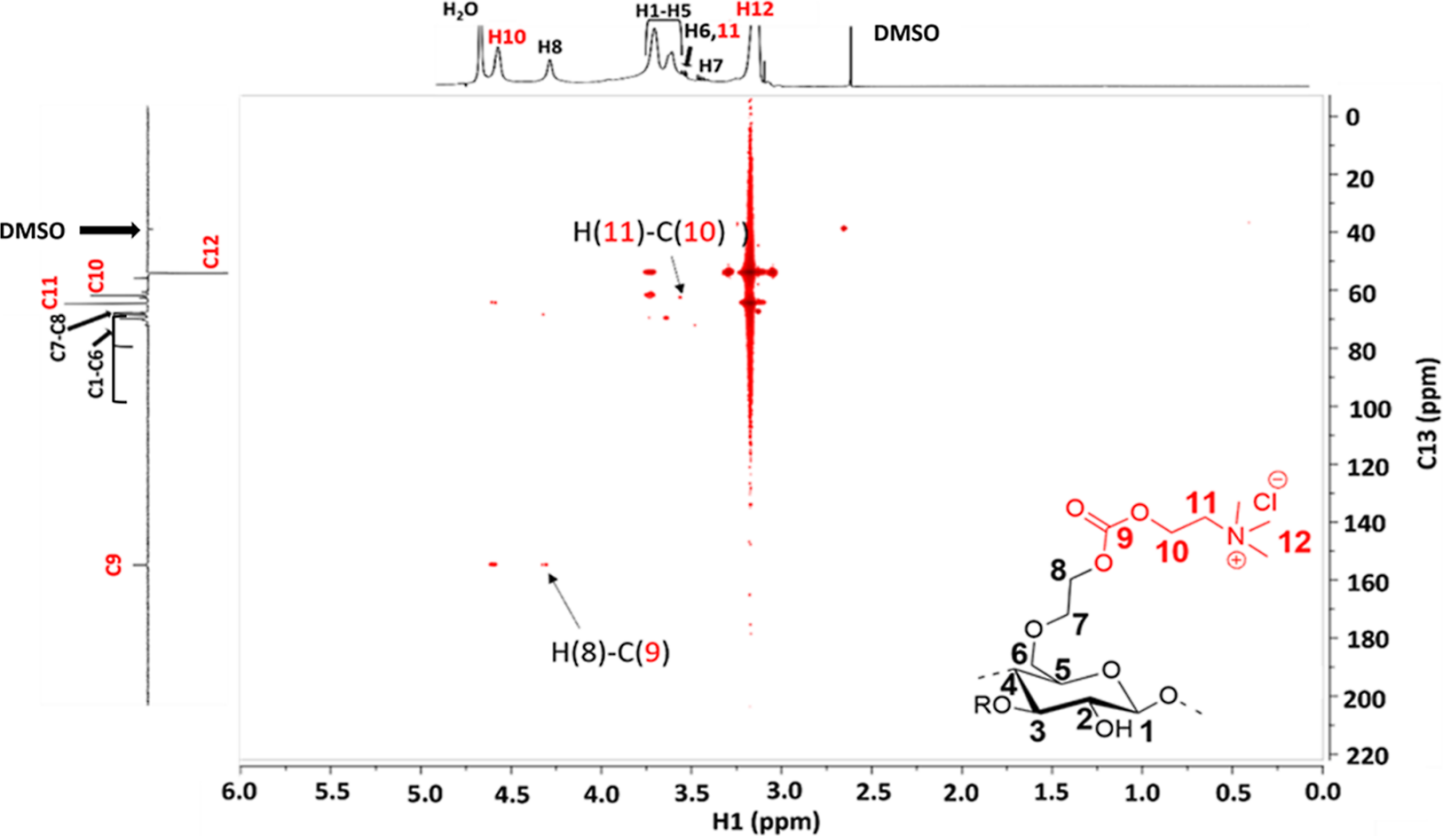
^1^H–^13^C HMBC spectra of c-HEC.

Peaks of carbons of the ethoxy group of HEC can be observed between δ 63 and δ 75 ppm. Comparing the proton spectra of c-HEC (peak 10 at Figure S14) with the proton of the CH_2_ group of Ch-Cl (peak 10 at Figure S4), there is a visible downfield shift of protons of the covalently bound cationic material, which is caused by the deshielding effect of the nearby electronegative oxygen of the carbonate ester. The peak at δ 3.5 ppm, which is attributed to the protons of the quaternary ammonium group, has a higher intensity for c-HEC and then for q-HEC (Figure S16). This can be explained by the DS, which is lower for q-HEC than for c-HEC. ^1^H–^13^C correlation signals observed in HSQC (Figure S20) allows identifying directly connected H–C pairs. With the ^1^H–^13^C HMBC spectra of c-HEC (Figure 3), more information was obtained regarding the covalently bound choline. Correlation between neighboring CH- groups were seen (H (11)–C (10)), but particularly, correlations between H(8)–C(9) show a covalent bonding between HEC and Ch–Cl. The presence of C(9) can be attributed to the carbonate ester of choline or to carbonate cross-links formed with other HEC hydroxyls.

### 3.3 Polymer Recovery Rates and Charge Density

The polymer recovery rates are 55 wt % for b-HEC, 85 wt % for b-PVA, and 59 wt % for c-HEC. A lower percentage of recovered polymers can be attributed to several steps of purification. Precipitation in acetone and several washes can lead to a loss of the product. Although all polymers come with a specified molecular weight, it will have a relatively broad molecular mass distribution,^26^ containing low molecular short polymer chains. Several days of dialysis in the dialysis tube with a 12–14 kDa cut-off can be the cause of the loss of polymer with these short chains.

A difference in the amount of nitrogen and consequently DS can be observed for all obtained polymers (Table 1). The highest amount of bound cationic charge was found for b-PVA, whereas b-HEC and c-HEC have half of the charge density per mass. Overall, all materials are highly charged strong polyelectrolytes with approximately twice the DS of commercial q-HEC.

**Table 1:** Elemental Composition, Cationic Charge from Polyelectrolyte Titration, and Calculated DS of b-HEC, b-PVA, c-HEC, and q-HEC^a^

	% N	% C	% H	charge (PE) (mmol/g)	DS_EA_	DS_PE_
betaine HCl_theor._	9.12	39.10	7.87	9.51		
choline chloride_theor._	10.03	43.01	10.11	7.16		
HEC_theor._		47.28	7.11			
PVA_theor._		54.53	9.15			
b-HEC	3.72 ± 0.02	36.29 ± 0.31	6.45 ± 0.03	2.27 ± 0.37	1.22	1
b-PVA	5.76 ± 0.06	42.79 ± 0.03	8.48 ± 0.13	4.46 ± 1.22	0.41	0.68
c-HEC	2.94 ± 0.03	29.36 ± 0.35	5.27 ± 0.21	2.21 ± 0	0.94	1.03
q-HEC	1.46 ± 0.05	43.71 ± 0.14	7.69 ± 0.30	1.55 ± 0	0.36	0.56

a EA and charge of starting materials was calculated from theory.

The flocculation capacity of the derivatives and the unmodified celluloses are depicted in Figure S23. With a higher amount of added cationic material, there is a higher transmittance, which means better flocculation. This can be explained by the neutralization of the negatively charged surface of kaolin and the formation of aggregates.^24^

A high increase of the transmittance is seen for q-HEC, c-HEC, and b-PVA, starting at 0.5 mg/mL, after it reaches a plateau at 2 mg/mL. b-HEC shows a lower flocculation capacity with a plateau at 70% at a concentration of 4 mg/mL, a fact that cannot be explained by the charge density. Unmodified HEC does not significantly influence the stability of the kaolin suspension, which indirectly confirms the presence of cationic charges in the products.

### 3.4 Biological Study

#### 3.4.1 Biocompatibility

**Figure 4:**
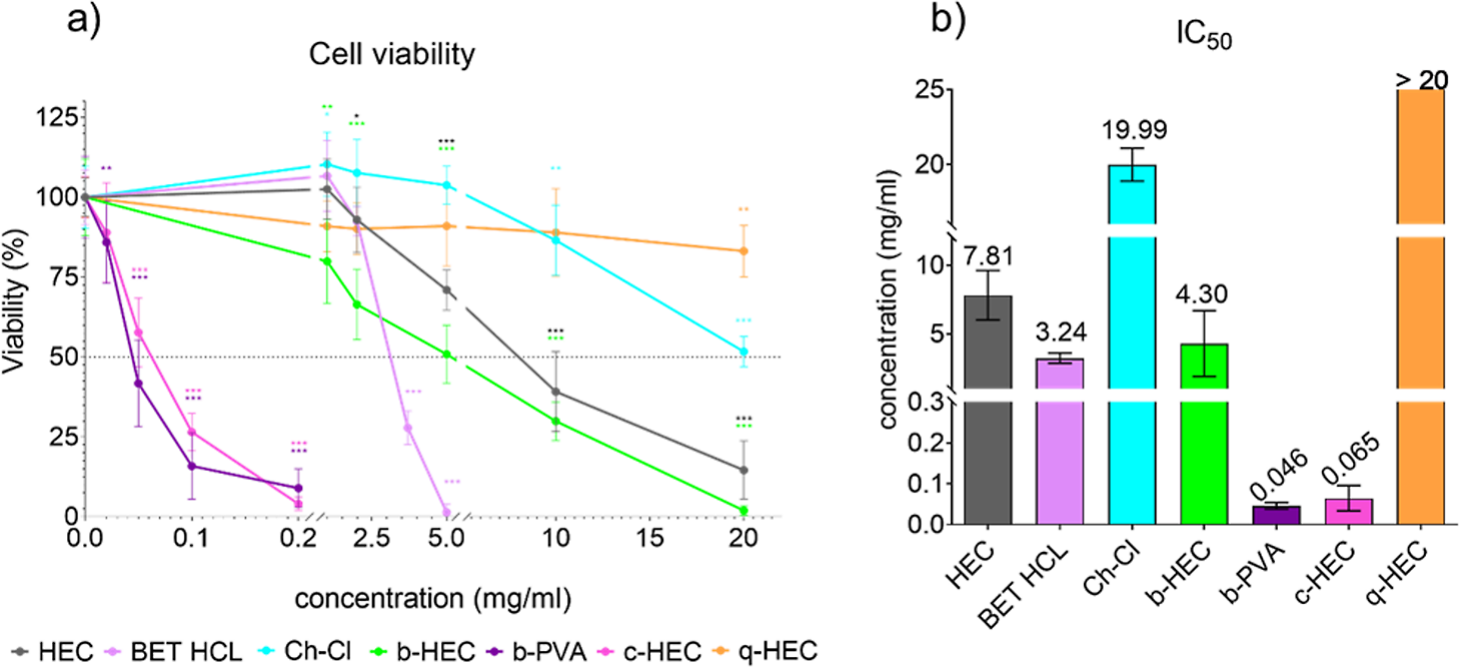
Cytotoxicity of cationized derivatives of HEC and PVA. (a) Changes in cell viability after 24 h of exposure of L929 cells to cationized derivatives of HEC and PVA. Data are presented as percentage of the negative control (complete growth medium). The asterisks denote statistically significant difference to the negative control (ANOVA and Dunnet’s multiple comparison test; *p < 0.05, **p < 0.01, and ***p < 0.001); (b) IC_50_ values for cationized derivatives of HEC and PVA in the L929 cell line. The positive control 5% DMSO elicited expected responses in all performed MTT tests—it decreased the cell viability to 11.1 ± 4.1% after 24 h of exposure (data not shown), confirming the validity of the tests.

**Figure 5:**
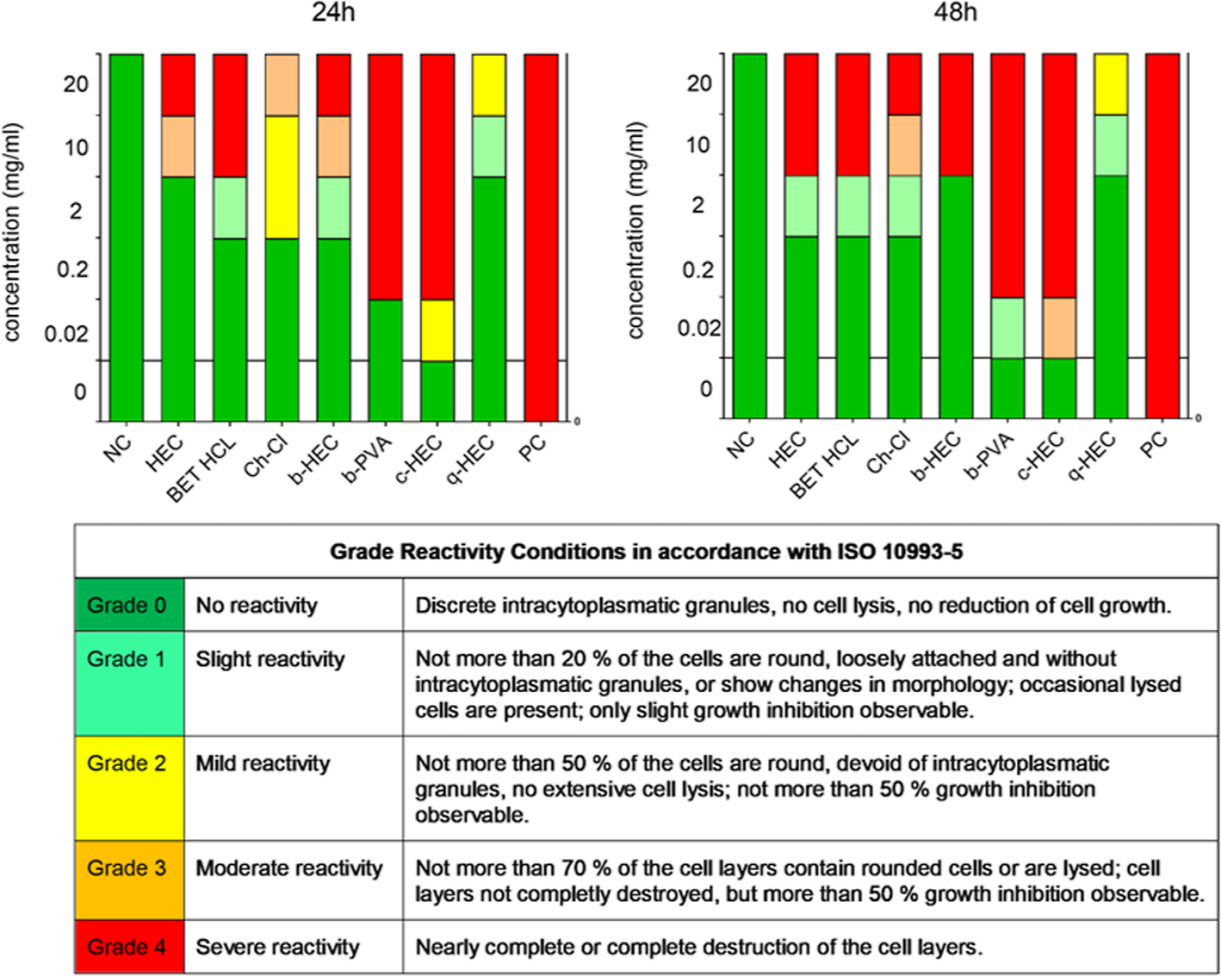
Biological reactivity of cationized derivatives of HEC and PVA after 24 h of exposure (left) and after 48 h of exposure (right) in the L929 cell line. Morphological changes were evaluated under the light microscope and graded according to ISO 10993-5. NC is the negative control (complete growth medium) and PC is the positive control (5% DMSO). The controls were tested at only one concentration, and a uniform bar is shown for visualization.

The biocompatibility of the cationic derivatives was evaluated through assessment of cytotoxicity and biological reactivity of the samples against mouse fibroblasts L929 in accordance with ISO 10993-5. Cationization of HEC with choline and of PVA with betaine significantly decreased L929 cell viability (Figure 4) and caused biological reactivity (Figure 5), while commercial q-HEC did not affect cell viability, at the tested concentrations (with an indeterminable IC_50_ of well above 20 mg/mL), highly charged b-PVA, and especially c-HEC were much more cytotoxic to L929 cells. Interestingly, the cytotoxicity of c-HEC (IC_50_ = 0.065 ± 0.031 mg/mL) was much higher than that of HEC (IC_50_ = 7.81 ± 1.80 mg/mL). Monomeric Ch-Cl was much less cytotoxic (IC_50_ of 19.99 ± 1.09 mg/mL) than BET HCl which can also be attributed to the acidity of the latter. An increase in cytotoxicity after binding to the polymer can be explained by the increased molecular weight and number of charges per molecule that could lead to increased interaction with phospholipid membranes in contrast to unbound Ch-Cl.^27−29^

Traces of imidazole or *O*-acyl-imidazolide on the polymer backbone (Scheme 2) could be detected by NMR (aromatic signals) but not by IR spectroscopy. Further studies are needed to clarify the mechanism of cytotoxicity, especially in the case of c-HEC and b-PVA. The likelihood that imidazolide structures can influence cytotoxicity despite their low concentration cannot be fully excluded and must therefore be a part of these studies.^30^

The cytotoxicity of unmodified PVA could not be determined in the tested concentration range due to its insolubility in the cell growth media. It can therefore not be stated whether cationization of PVA affects its cytotoxicity. It can however be concluded that b-PVA was much more cytotoxic than BET HCl, with a more than 70-fold lower IC_50_. According to literature data, the IC_50_ value of PVA for L929 cells is higher than 4 mg/mL^31^ or even higher than 10 mg/mL,^32^ which means that the covalent binding of BET HCl to PVA significantly increased its cytotoxicity. It is important to note that the MEM cell media (native pH value: 7.3) do not provide a large pH-buffering capacity due to its ingredience. The pH value of the polymers dissolved in the media without cells was therefore measured and found to be b-PVA, 2 mg/mL, pH: 8.4; q-HEC, 20 mg/mL, pH: 8.1. It can be concluded that a slight increase in the pH value of the cell media due to the polymers is not the main cause for the high toxicity of b-PVA since unmodified HEC is the least toxic but also slightly alkaline. Nevertheless, as stated above, the presence of basic imidazole could also contribute to cytotoxicity.

Representative light microscopy images of the cells are shown in Figures S24–S30. The biological reactivity was evaluated by visual inspection of morphological changes under the light microscope. The changes were graded on a scale 0 - 4, which are summarized and explained in Figure 5. The observations correlate with the results of the cytotoxicity tests. At low concentrations, all samples were rated with no reactivity, slight or mild reactivity (grades 0–2). Higher concentrations were rated with a moderate or severe reactivity (grade 3 or 4). After 48 h of exposure, the observed morphological changes of the cells were generally similar or slightly enhanced compared to 24 h of exposure. It can be concluded that both cationic derivatives (b-PVA and c-HEC) are harmful for the fibroblasts whereas commercial q-HEC and b-HEC can be considered non-problematic at the concentrations investigated. It is not possible to draw conclusions about the molecular mechanism involved in the cytotoxic effects observed, and more in depth studies are necessary. Charge complexation will of course play the major but not the only role since c-HEC and b-HEC have similar charges per mass of product (Table 1). The strength of the electrostatic interaction however appears lower for b-HEC as can also be seen from the flocculation experiments (Figure S23). It is also very likely that the hydrolytic stability of the ester bonds in b-HEC is lower, leading to a cleave during the 24/48 h cell testing experiments. This stability must also be considered in potential applications of the materials but could also be an advantage, e.g., for constructing reversible charge complexes for drug delivery etc.

#### 3.4.2 Antimicrobial Activity

Quaternary ammonium compounds are known to have strong antimicrobial properties.^33^ Numerous publications have reported the antimicrobial activity of cationic macromolecules.^6,34,35^Table 2 summarizes the MIC and MBC results for both bacterial strains and all compounds tested. Tables S1 and S2 show the concentration-dependent effects on the growth/inhibition of both strains. BET HCl showed an inhibition of growth of both strains at a concentration of 5 mg/mL (*S. aureus*) and 2.5 mg/mL (*P. aeruginosa*). In the case of b-PVA and *S. aureus*, the results from the broth dilution test (liquid media) were not showing a clear concentration dependence (Table S1). The polymer solutions were therefore applied to solid media and the growth assessed visually. No inhibition of *S. aureus* could be observed for b-PVA on the solid media, and a MIC > 20 mg/mL was therefore determined. For c-HEC, a strong growth inhibition of *S. aureus* in the dilution test was already observed at low concentrations (MIC, 0.08 mg/mL, and Table S1). The polymer solutions were therefore also applied to solid media containing the bacteria and a partial growth inhibition could be determined.

**Table 2:** Antimicrobial Activity of Betaine Hydrochloride, b-HEC, and b-PVA against *S. aureus* and *P. aeruginosa*

	S. aureus		P. aeruginosa	
	MIC (mg/mL)	MBC (mg/mL)	MIC (mg/mL)	MBC (mg/mL)
BET HCl	5	20	2.5	10
Ch-Cl	>20		>20	
b-PVA	>20		>20	
b-HEC	>20		>20	
c-HEC	0.08	>0.31	>10	
q-HEC	>5		>5	

The present work shows that betainates of HEC and PVA are not strong antimicrobials which supports the results of Holappa et al.^35^ b-HEC, q-HEC, and c-HEC have very similar polymeric backbones but different substituents, and the antimicrobial activity should depend on the DS of cationic charge. Commercial q-HEC however does not show any inhibition up to a concentration of the tested 5 mg/mL (Tables S1 and S2). Only c-HEC showed significantly increased antimicrobial activity and selectivity against *S. aureus* at lower concentrations. Since *P. aeruginosa* contains an outer cell membrane surrounding the cell wall (Gram negative), this strain is probably less sensitive to inactivation through c-HEC.^36^

## 4 Conclusions

Cationization of hydroxyethyl cellulose (HEC) and polyvinyl alcohol (PVA) with betaine or choline was achieved using carbonyl diimidazole (CDI) as an activation agent in DMSO. It was possible to either activate the polymers’ hydroxyls with an excess of CDI, followed by coupling of choline chloride, or to activate the betaine separately, and couple it to the polymers. The first approach led to the cross-linking of PVA, probably due to a different reactivity of the solely secondary hydroxyls present compared to HEC, where no cross-linking was observed. The covalent bonds between the polymers and the small molecules were confirmed in detail by 2D NMR and IR. All derivatives and commercial cationic HEC are good flocculants for kaolin and form charge complexes with an anionic polyelectrolyte, confirming the presence of cationic charge on the backbone. The cationic betaine esters of PVA (b-PVA) and choline esters of HEC (c-HEC) were cytotoxic for L929 fibroblasts. The reason for the high cytotoxicity compared to the betaine ester of HEC (b-HEC) and unmodified or commercial cationic HEC (q-HEC) is probably due to the amount of charge but also due to the peculiar properties of the choline substituent, which is also a cell metabolite and a part of many phospholipids. b-HEC and b-PVA did not show antimicrobial activity against *S. aureus* and *P. aeruginosa*. In contrast, the choline derivative c-HEC efficiently inhibited the growth of *S. aureus* (MIC 0.08 mg/mL) but less so of *P. aeruginosa*. It is therefore concluded that the choline polymer distinctively interacts with human and bacterial cells, probably through cell signaling or uptake. This hypothesis however requires further investigation of a wider variation of polymers bearing choline substituents that are potentially reactive toward living cells. Care must be taken to further investigate the role of (acyl-)imidazole as a coupling agent or residual substituent since many antimicrobials contain the imidazole structure which could interfere with bacteria and human cells. The ester bonds in the polymers might be relatively labile and susceptible to biodegradation by esterases, whereas the choline polymers could also inhibit (acetylcholine) esterases. In future, this stability should be assessed in detail. It is finally important to note that an application of the polymers for instance as antimicrobials in contact with human cells can be limited by the potential cytotoxic effects. However, the materials could be used, for instance, as anti-microbial coatings of solids not directly in contact with human tissue. The efficacy of such coatings could be evaluated in a follow-up work. More detailed studies on the environmental impact of the polymers would also be of interest in future works, for instance, by using a model organism such as a zebra fish.

## Supporting Information Available

Reaction scheme of betaine HCl with CDI, ATR-IR spectra, ^1^H, and ^13^C NMR spectra of starting materials (betaine hydrochloride, choline chloride, and 2-hydroxyethyl cellulose) and b-HEC, b-PVA, q-HEC, and c-HEC; ^1^H–^13^C NMR spectra of b-HEC, b-PVA, and c-HEC; flocculation performance of the polymers with kaolin, light microscopy images of morphology of the L929 mouse fibroblasts cells after exposure to substances; and concentration-dependent antimicrobial properties of the substances (PDF)
