# Supplementary material for: Cytotoxicity and Antibacterial Efficacy of Betaine- and Choline-Substituted Polymers
Source: ACS Appl Polym Mater. 2023 Jun 13;5(7):5270–9. doi: 10.1021/acsapm.3c00691 (PMC10353005; doi:10.1021/acsapm.3c00691)
Supplement: Supplementary file 1 — ap3c00691_si_001.pdf [file ap3c00691_si_001.pdf]

Supporting Information

Cytotoxicity and antibacterial efficacy of betaine and choline substituted polymers

Lucija Jurko[1], Damjan Makuc[2], Alja Štern[3], Janez Plavec[2,4,5], Bojana Žegura[3], Perica Bošković[6], Rupert Kargl*[1,7]

[1]Laboratory for Characterization and Processing of Polymers (LCPP), Faculty of Mechanical Engineering, University of Maribor, Smetanova ulica 17, SI - 2000 Maribor, Slovenia

[2]Slovenian NMR Centre, National Institute of Chemistry, Hajdrihova 19, SI-1000 Ljubljana, Slovenia

[3]Department of Genetic Toxicology and Cancer Biology, National Institute of Biology, Večna pot 111, 1000 Ljubljana, Slovenia

[4]EN-FIST Centre of Excellence, Trg Osvobodilne fronte 13, 1000 Ljubljana, Slovenia

[5]Faculty of Chemistry and Chemical Technology, University of Ljubljana, Večna pot 113, 1000 Ljubljana, Slovenia

[6]Department of Chemistry, Faculty of Science, University of Split, Ruđera Boškovića 33, 21000 Split, Croatia

[7] Institute for Chemistry and Technology of Biobased System, Graz University of Technology, Stremayrgasse 9, 8010 Graz, Austria

To whom correspondence should be addressed

rupert.kargl@tugraz.at

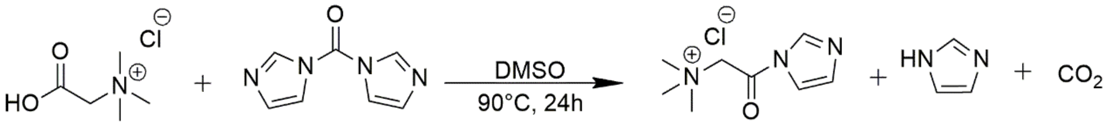

Scheme S1: *Reaction between betaine HCl and CDI forming N-acyl imidazole betainate in DMSO.*

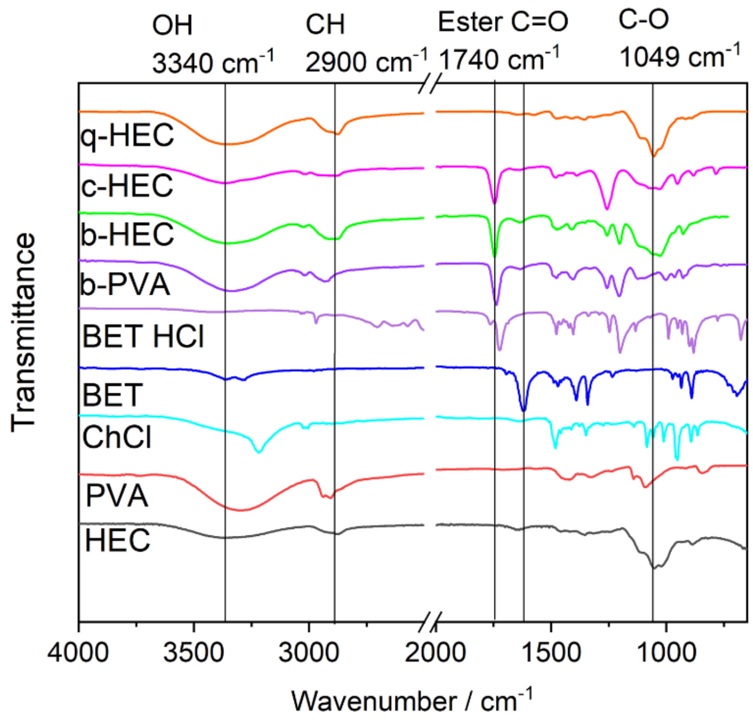

Figure S1: *FTIR spectra of betaine and choline esters of hydroxyethyl cellulose (b-HEC, c-HEC) and polyvinyl alcohol (b-PVA), of all starting materials, and of commercial cationic q-HEC.*

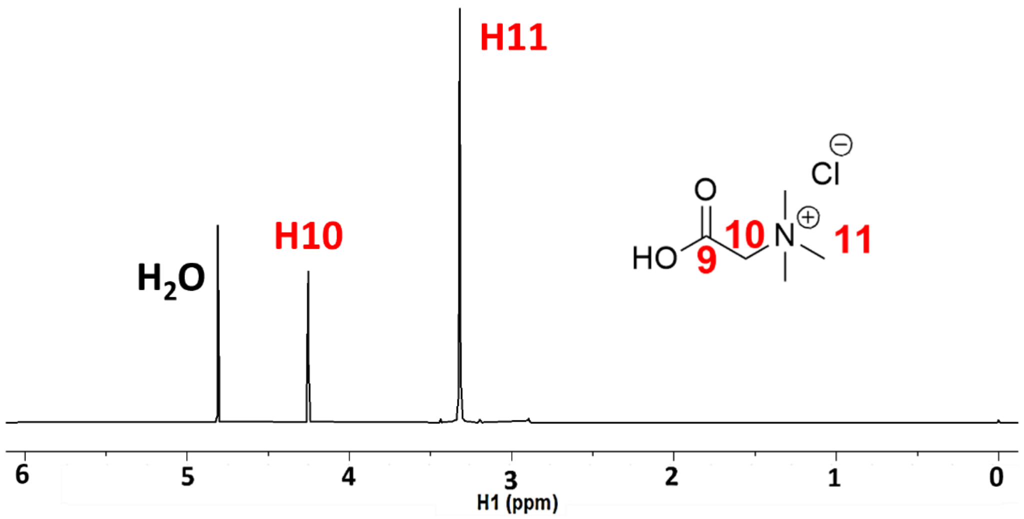

Figure S2: *^{1}H NMR of BET HCl*

BET HCl: ^{1}H NMR (600 MHz, D_2O) δ 3.31, 4.25.

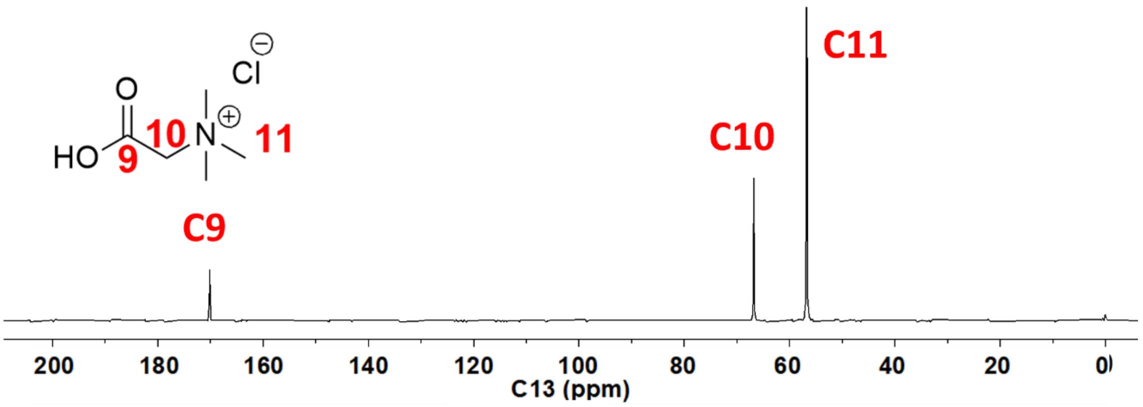

Figure S3: *^{13}C NMR of BET*

BET HCl: ^{13}C NMR (151 MHz, D_2O, δ in ppm TMS) δ 56.7, 66.8, 170.2.

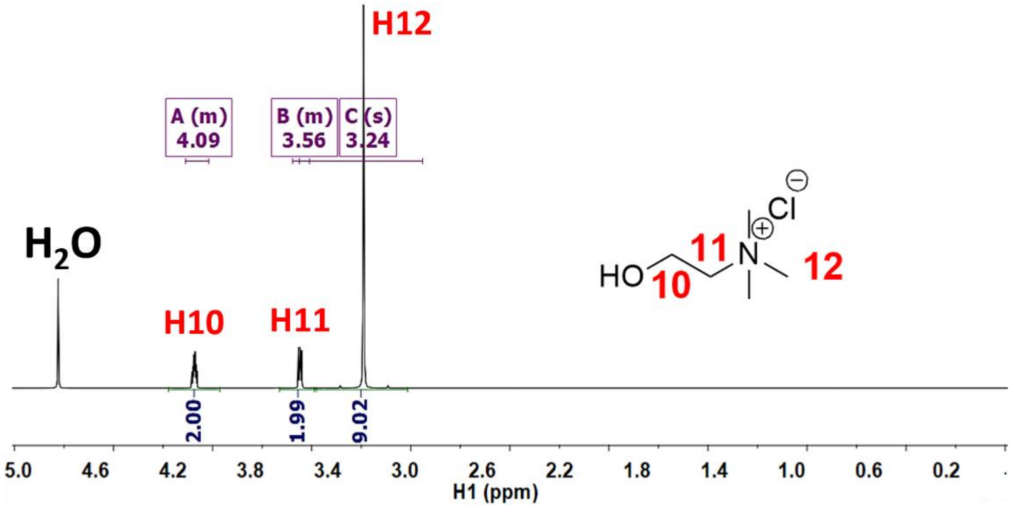

Figure S4: *^{1}H NMR of Ch-Cl*

Ch-Cl: ^{1}H NMR (600 MHz, D_2O) δ 3.24, 3.56, 4.09.

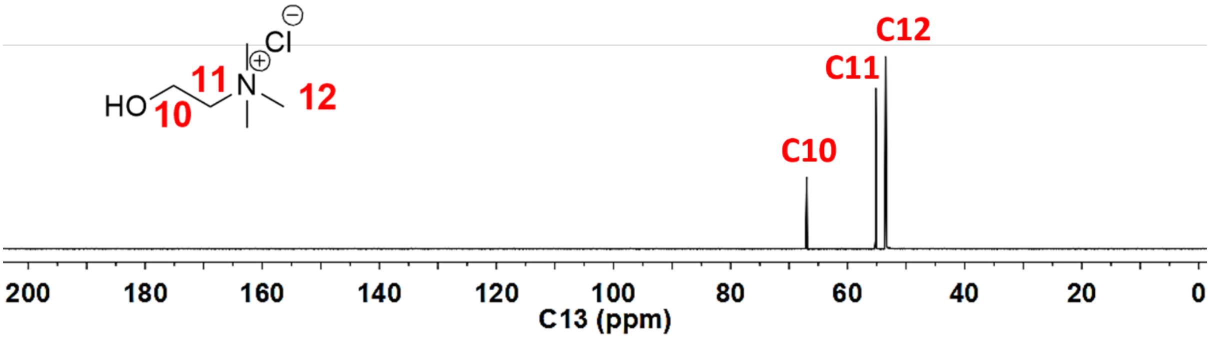

Figure S5: *^{13}C NMR of Ch-Cl*

Ch-Cl: ^{13}C NMR (151 MHz, D_2O) δ 53.5, 55.2, 67.0.

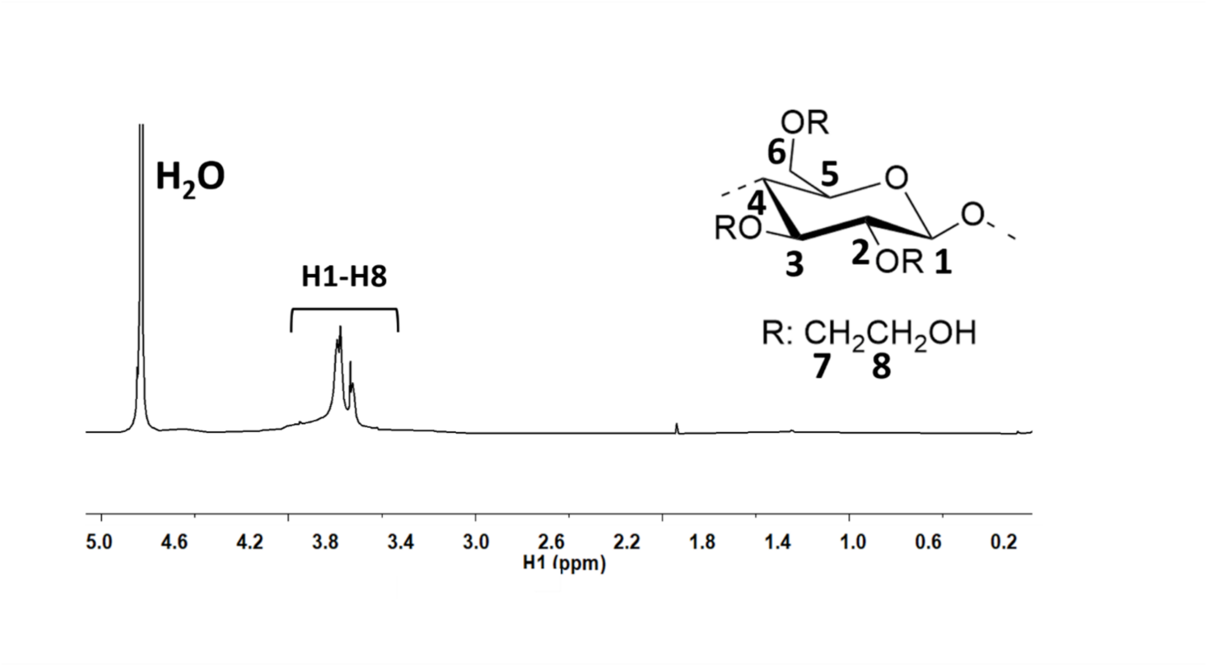

Figure S6: *^{1}H NMR of HEC*

HEC: ^{1}H NMR (600 MHz, D_2O, δ in ppm TMS) δ 3.6-4.0.

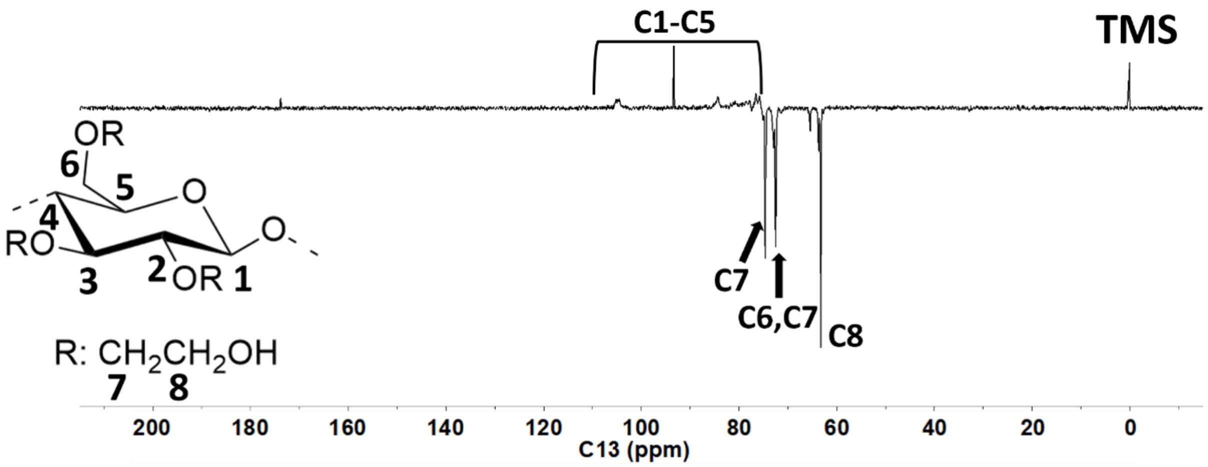

Figure S7: *^{13}C NMR of HEC*

HEC: ^{13}C NMR DEPTQ (151 MHz, D_2O) δ 63.3, 72.5, 74.6, 75.8, 76.6, 84.3, 104.5.

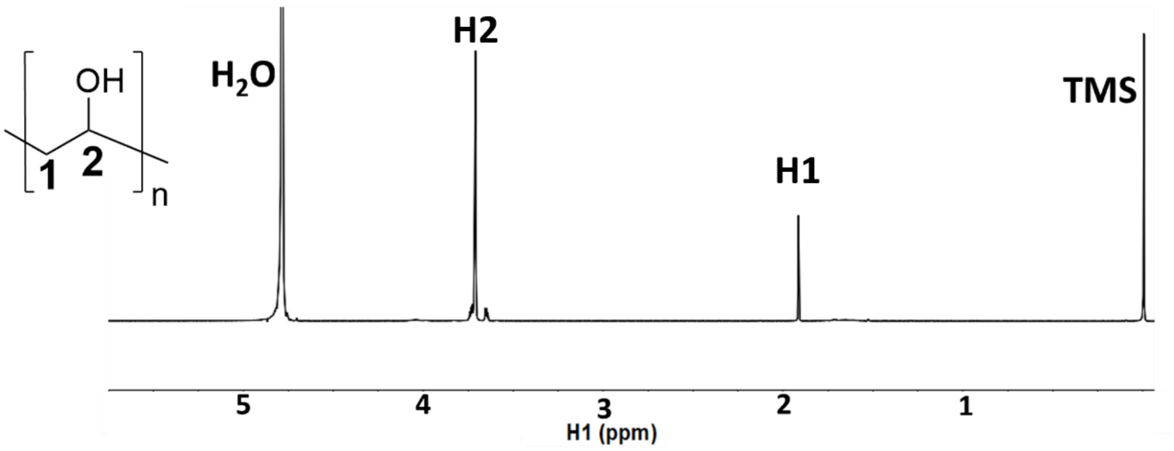

Figure S8: *^{1}H NMR of PVA*

PVA, 89-90% hydrolysed MW=89 000 g/mol ^{1}H NMR (600 MHz, D_2O) δ 1.92, 3.72.

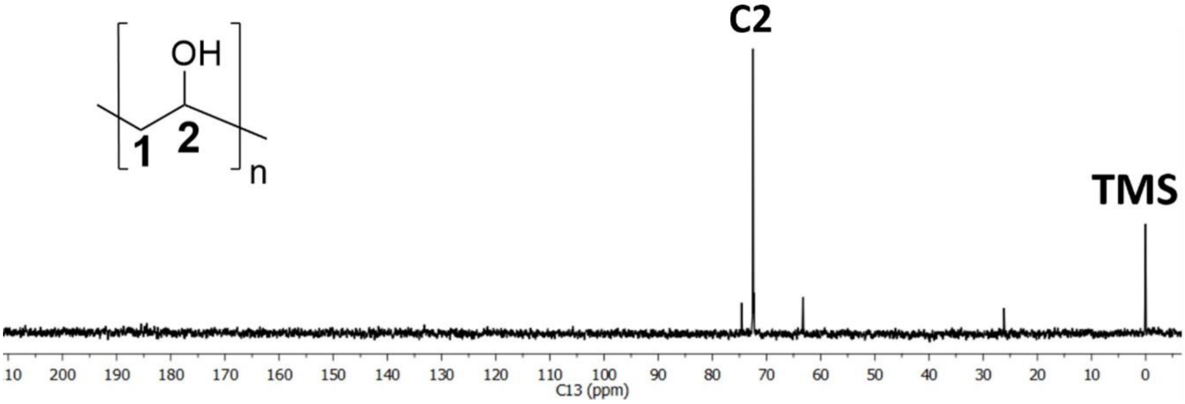

Figure S9: *^{13}C NMR of PVA*

PVA, 89-90% hydrolysed MW=89 000 g/mol: ^{13}C NMR (151 MHz, D_2O) δ 26.1, 63.2, 72.4, 74.6.

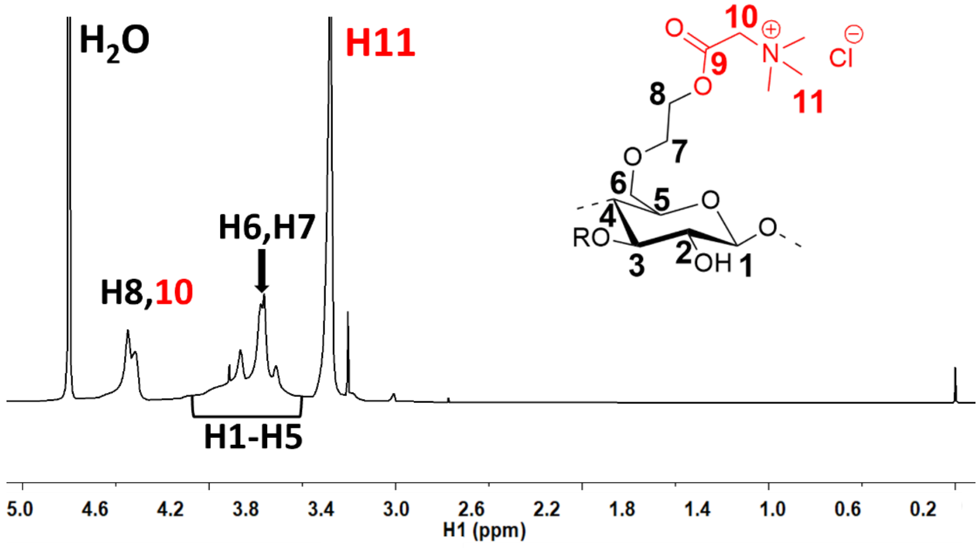

Figure S10: *^{1}H NMR of b-HEC*

b-HEC: ^{1}H NMR (600 MHz, D_2O, δ in ppm TMS) δ 3.3, 3.35, 3.64, 3.70, 3.71, 3.72, 3.83, 3.89, 4.39, 4.43.

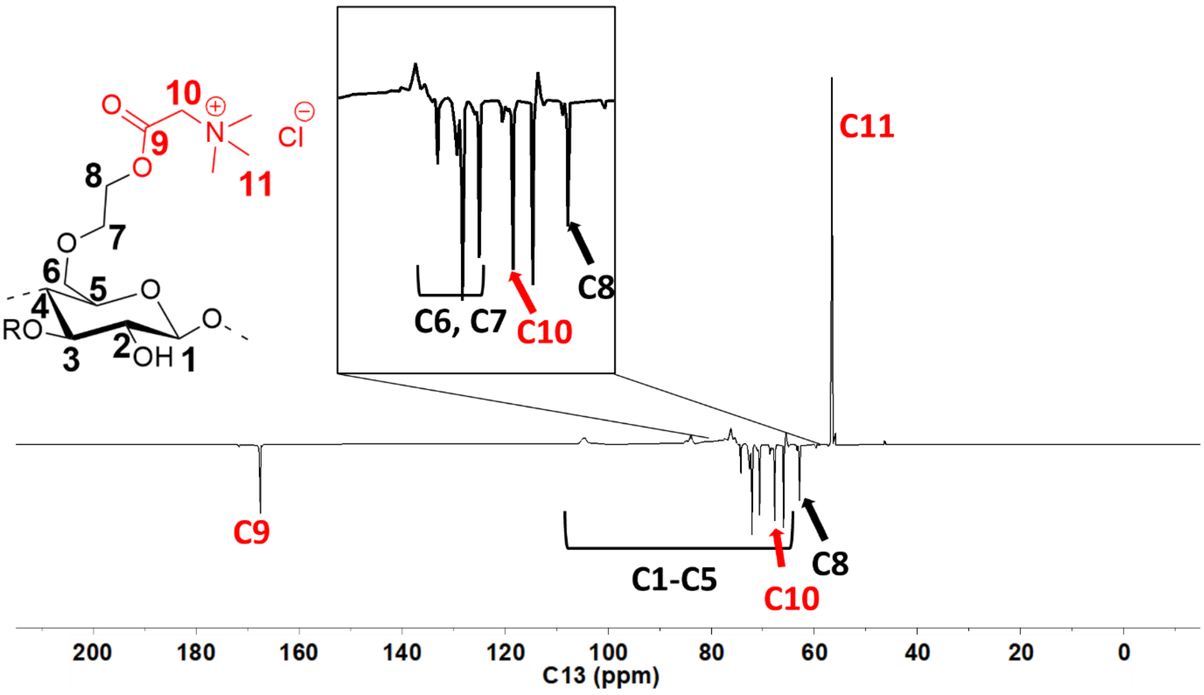

Figure S11: *^{13}C NMR of b-HEC*

b-HEC: ^{13}C NMR DEPTQ (151 MHz, D_2O) δ 55.9, 56.6, 62.9, 65.5, 65.9, 67.8, 70.7, 72.1, 72.6, 74.3, 75.4, 76.2, 77.5, 84.0, 104.6, 167.6.

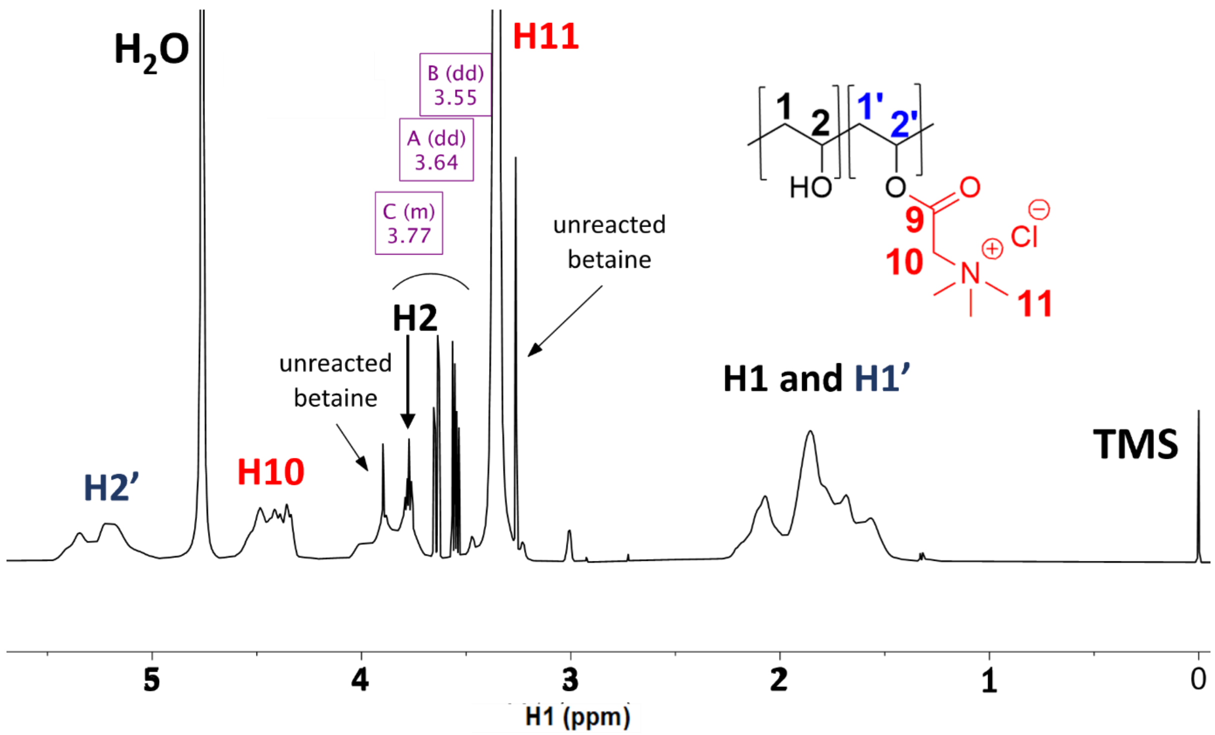

Figure S12: *^{1}H NMR of b-PVA*

b-PVA: ^{1}H NMR (600 MHz, D_2O, δ in ppm TMS) δ 1.57, 1.68, 1.86, 2.07, 3.00, 3.26, 3.35, 3.53, 3.55, 3.63,3.65, 3.72, 3.75, 3.77, 3.79, 3.88, 3.90 4.34, 4.39, 4.42, 5.22, 5.35.

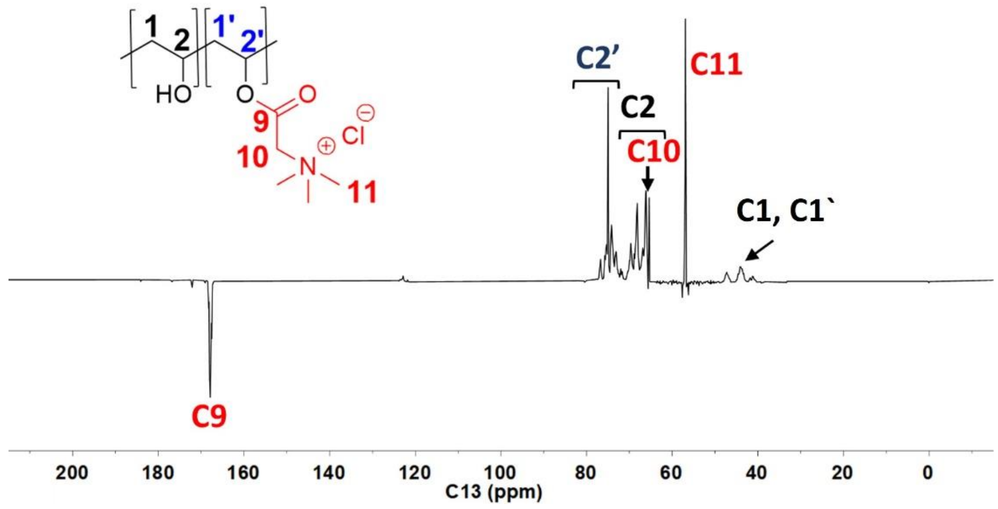

Figure S13: *^{13}C NMR of b-PVA*

b-PVA: ^{13}C NMR DEPTQ (151 MHz, D_2O, δ in ppm TMS) δ41.3, 41.8, 44.2, 47.3, 56.9, 65.4, 66.1, 66.8, 68.3, 69.7, 71.7, 72.0, 73.1, 74.2, 74.4, 75.0, 75.9, 75.8,76.7, 122.9, 167.5, 167.7, 167.9, 167.9, 168.1, 168.2, 172.1.

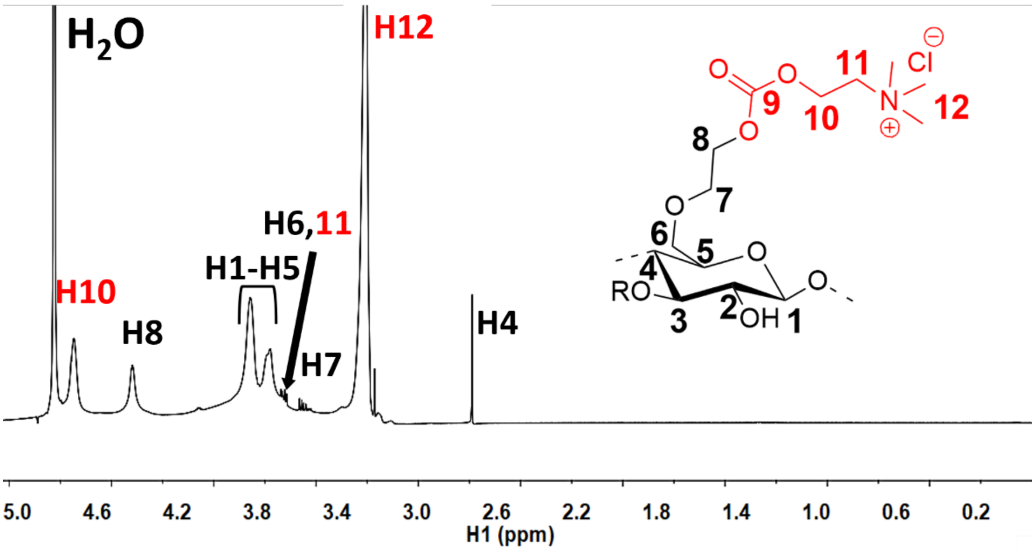

Figure S14: *^{1}H NMR of c-HEC*

c-HEC: ^{1}H NMR (600 MHz, D_2O, δ in ppm TMS) δ 4.78, 4.68, 4.40, 3.82, 3.74, 3.72, 3.26.

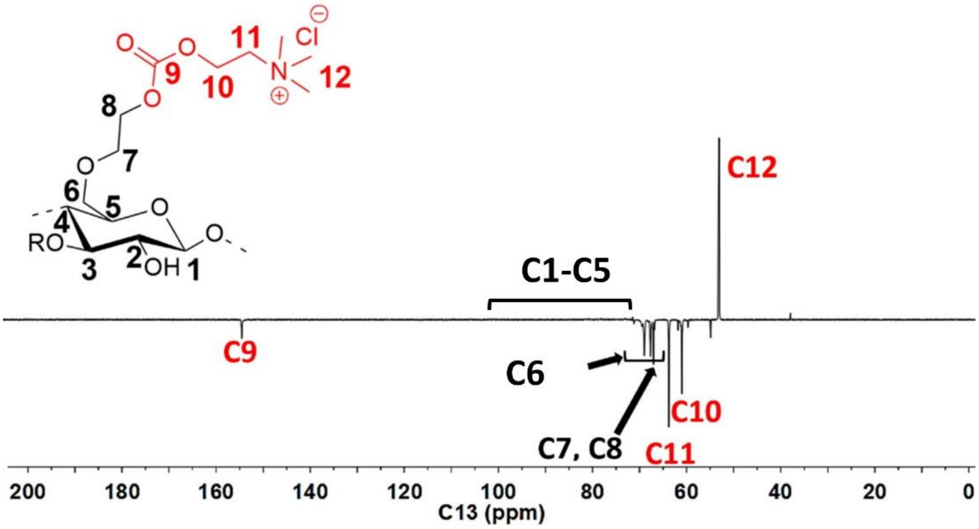

Figure S15: *^{13}C NMR of c-HEC*

c-HEC: ^{13}C NMR DEPTQ (151 MHz, D_2O, δ in ppm TMS) δ 154.60, 70.10, 69.64, 68.33, 67.65, 67.42, 64.45, 62.50, 61.95, 61.67, 60.42, 55.62, 53.86.

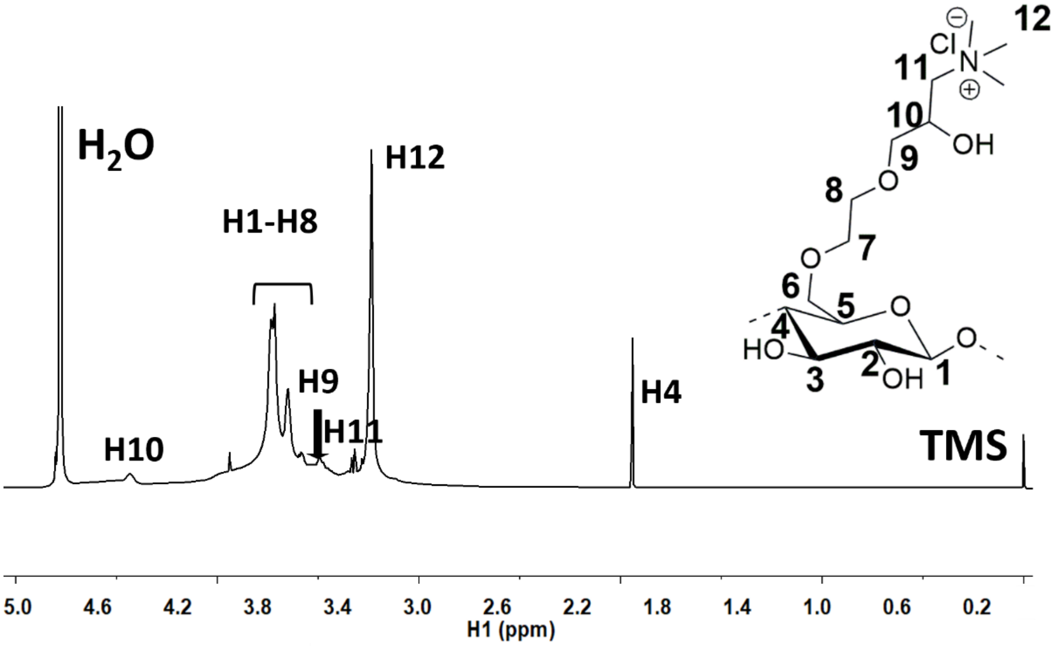

Figure S16: [1]*H NMR of q-HEC*

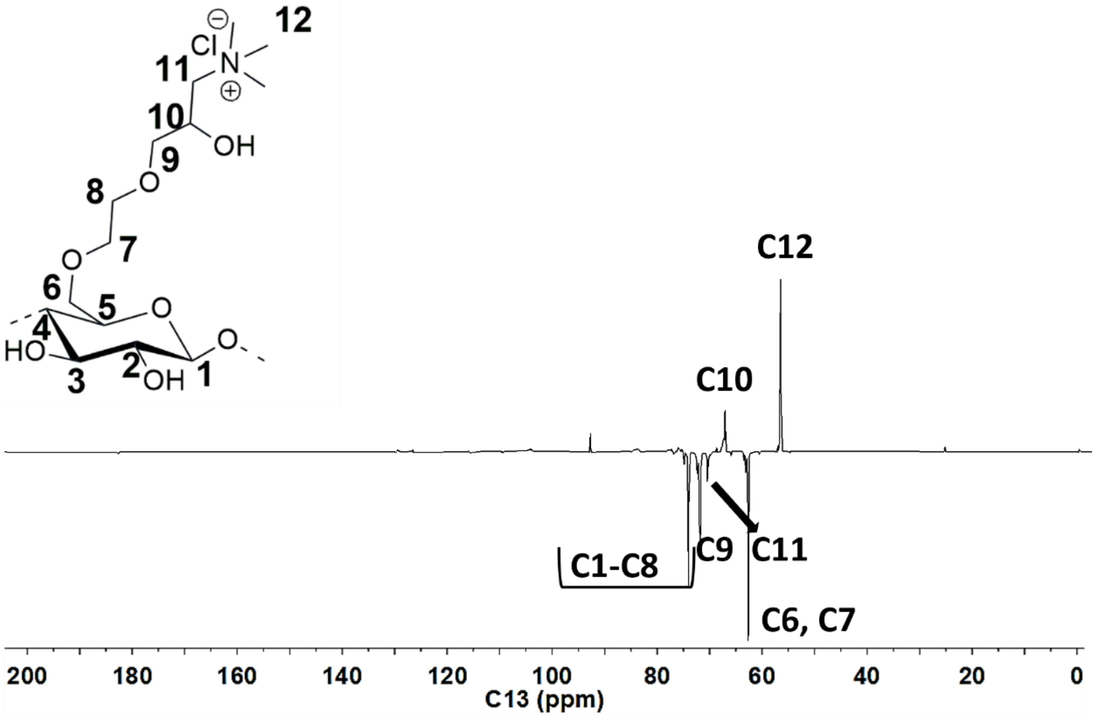

Figure S17: [13]*C DEPTQ NMR (151 MHz, D_2O) of q-HEC*

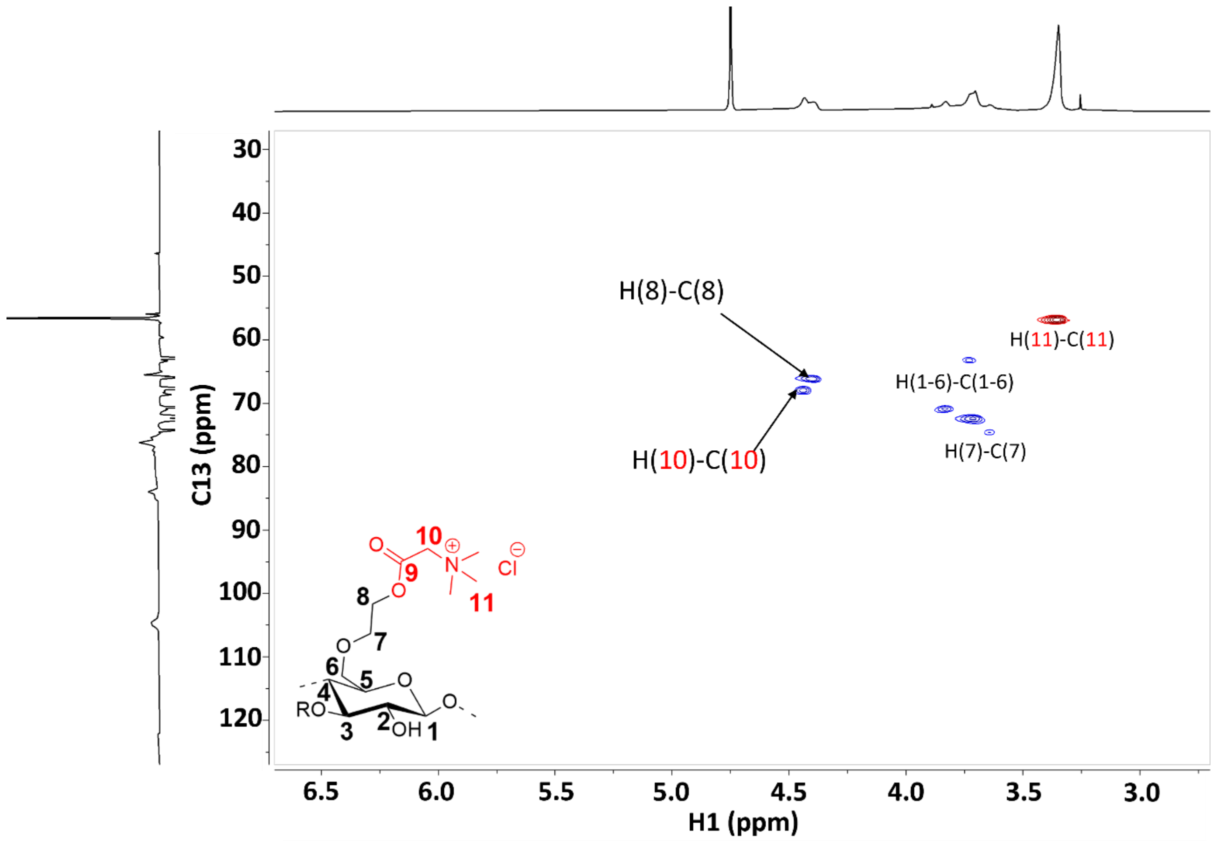

Figure S18: *^{1}H-^{13}C HSQC (D_2O) of b-HEC*

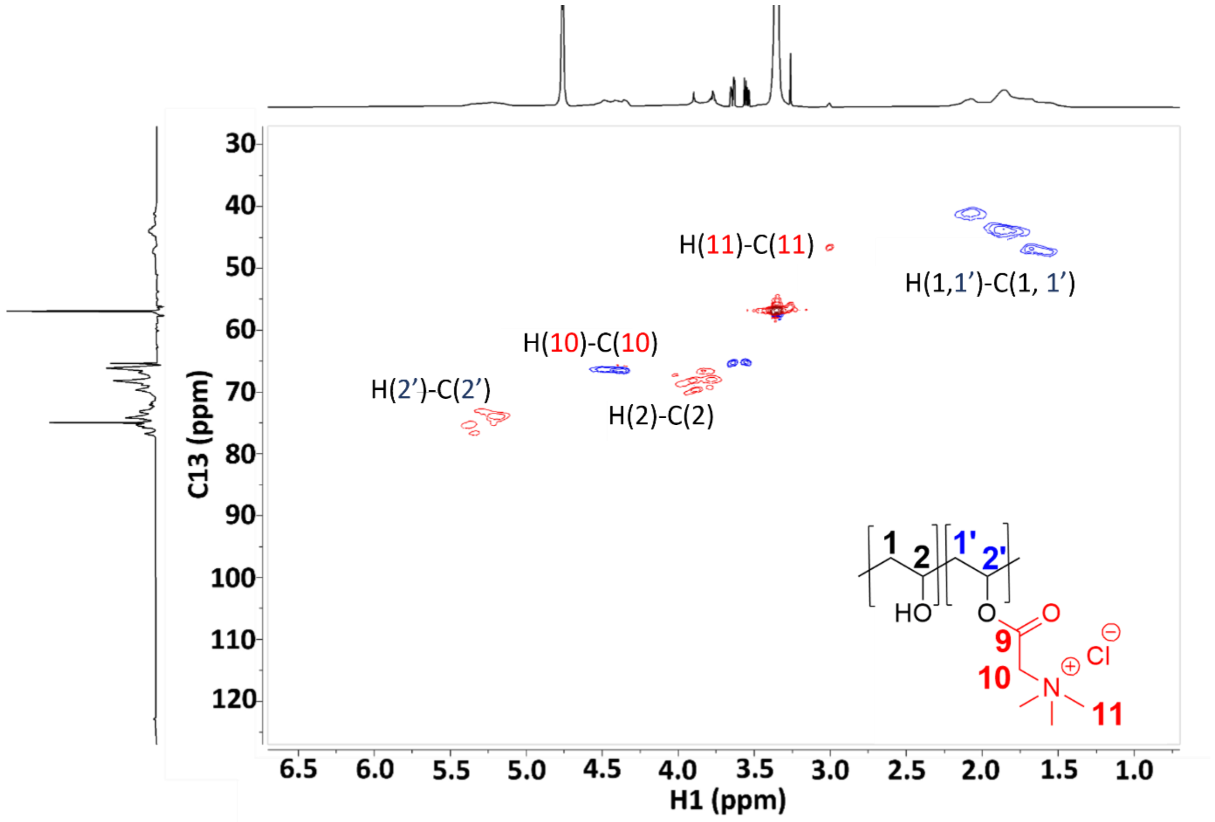

Figure S19: *^{1}H-^{13}C HSQC (D_2O) of b-PVA*

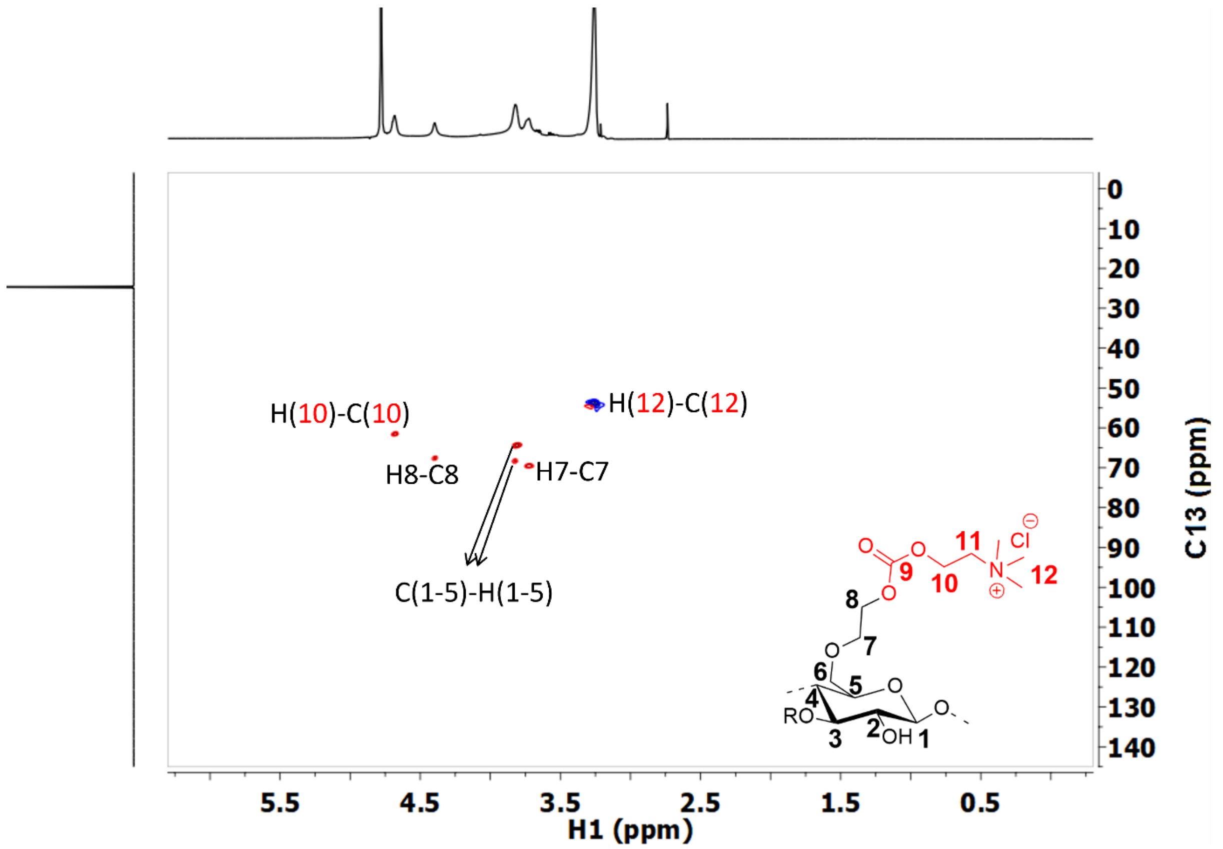

Figure S20 HSQC of c-HEC

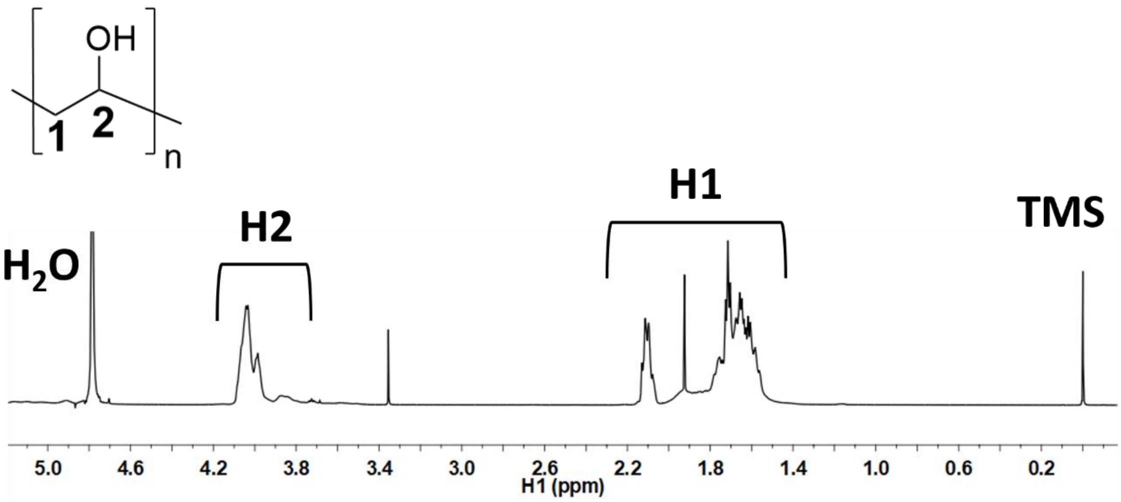

Figure S21: *^{1}H NMR of PVA, Mowiol® 8-88, Mw ~67,000 Da*

PVA ^{1}H NMR (600 MHz, D_2O) δ 4.79, 4.04, 3.99, 3.86, 3.73, 3.69, 3.35, 2.13, 2.11, 2.10, 2.08, 1.92, 1.76, 1.73, 1.71, 1.67, 1.66, 1.64, 1.62, 1.61, 1.59, 1.56. Residual acetyl groups present.

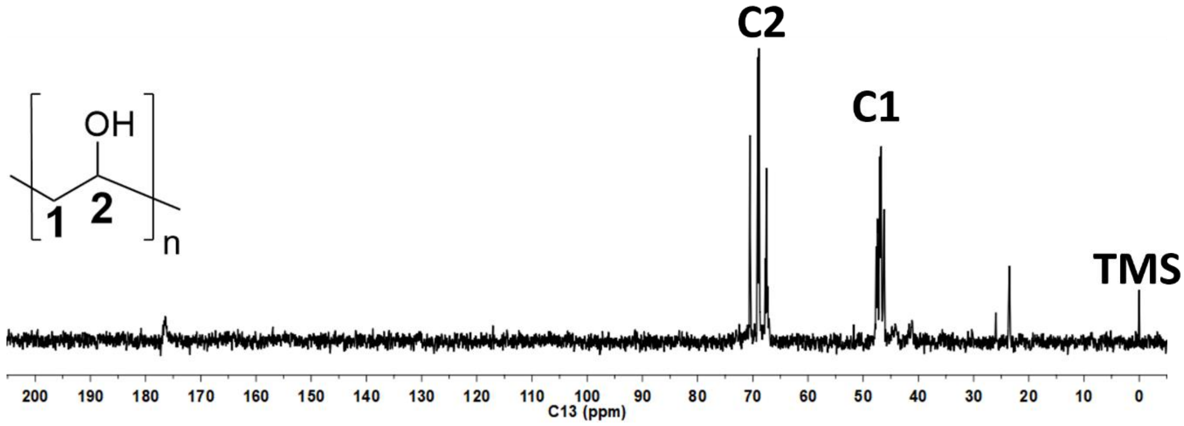

Figure S22: *^{13}C NMR of PVA, Mowiol® 8-88, Mw ~67,000 Da*

PVA: ^{13}C NMR (151 MHz, D_2O) δ 70.54, 69.10, 68.88, 67.74, 67.55, 67.36, 47.65, 47.40, 46.99, 46.76, 46.27. Residual acetyl groups present (23, 176).

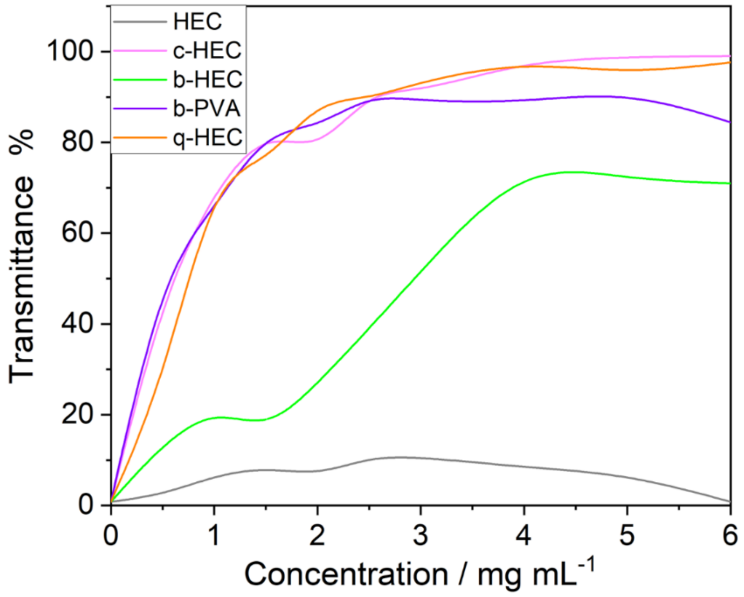

Figure S23: *Flocculation performance of HEC, b-HEC, q-HEC, b-PVA and c-HEC in a 0.25 wt% kaolin suspension*

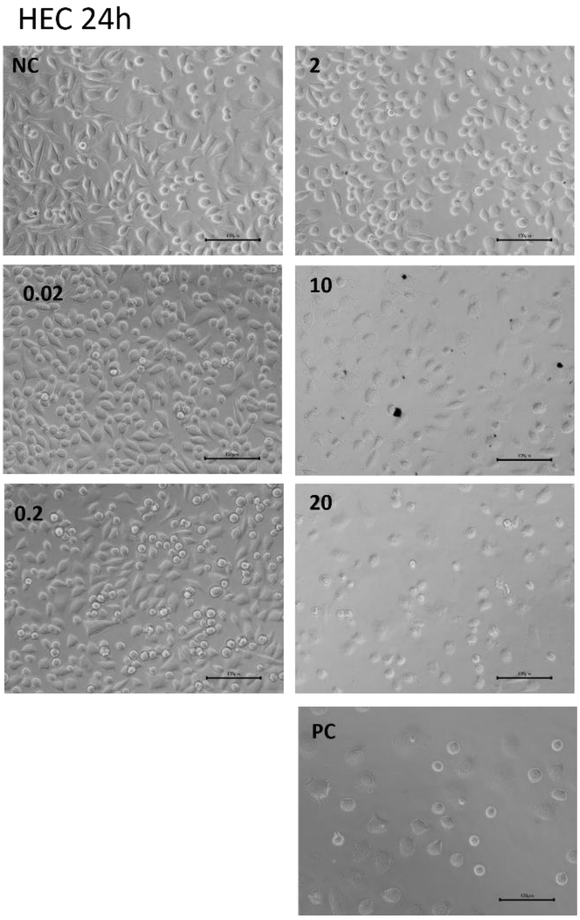

Figure S24: *Morphology of the L929 mouse fibroblasts cells after 24 h and 48 h exposure to HEC. NC negative control, PC positive control. The numbers indicate the concentration of HEC in mg/ml.*

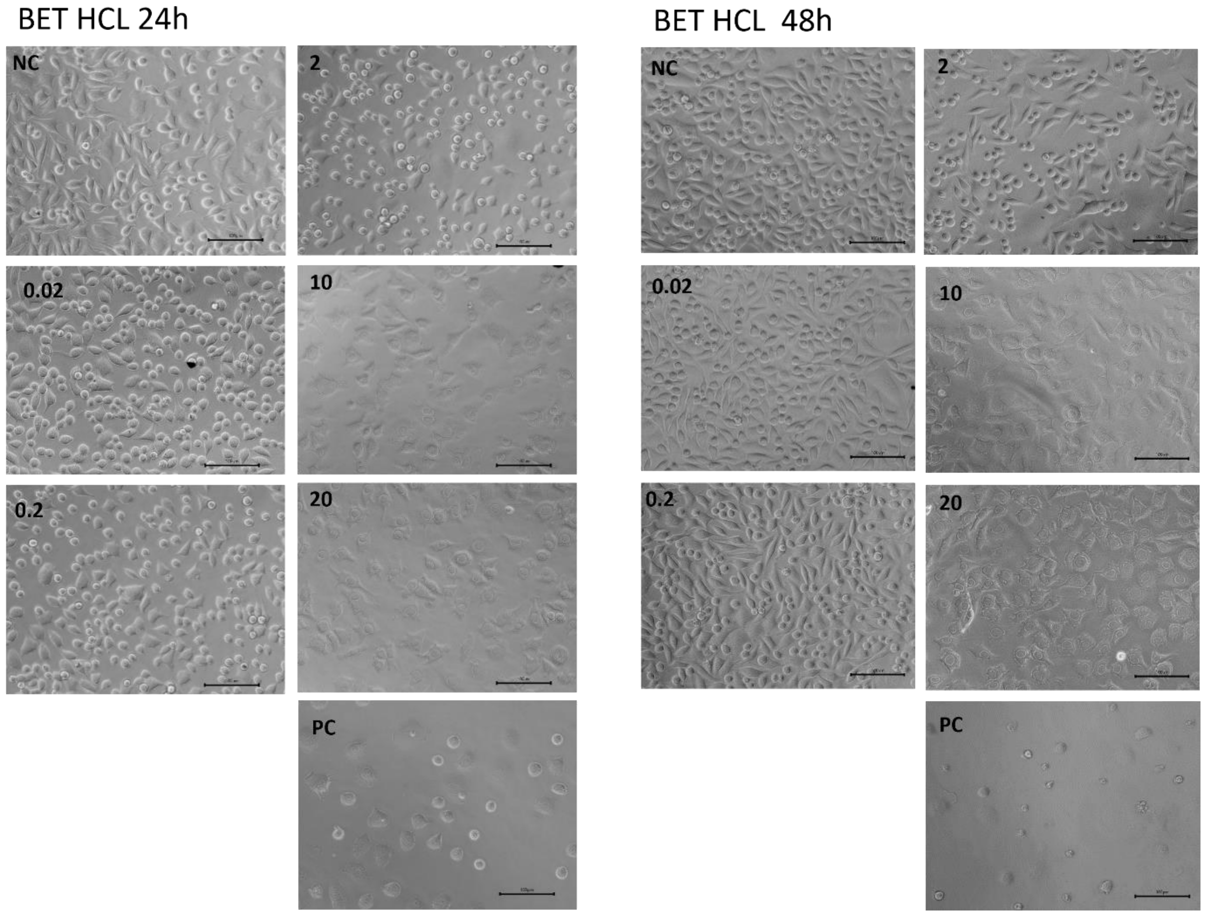

Figure S25: *Morphology of the L929 mouse fibroblasts cells after 24 h and 48 h exposure to BET HCl. NC negative control, PC positive control. The numbers indicate the concentration of BET HCl in mg/ml.*

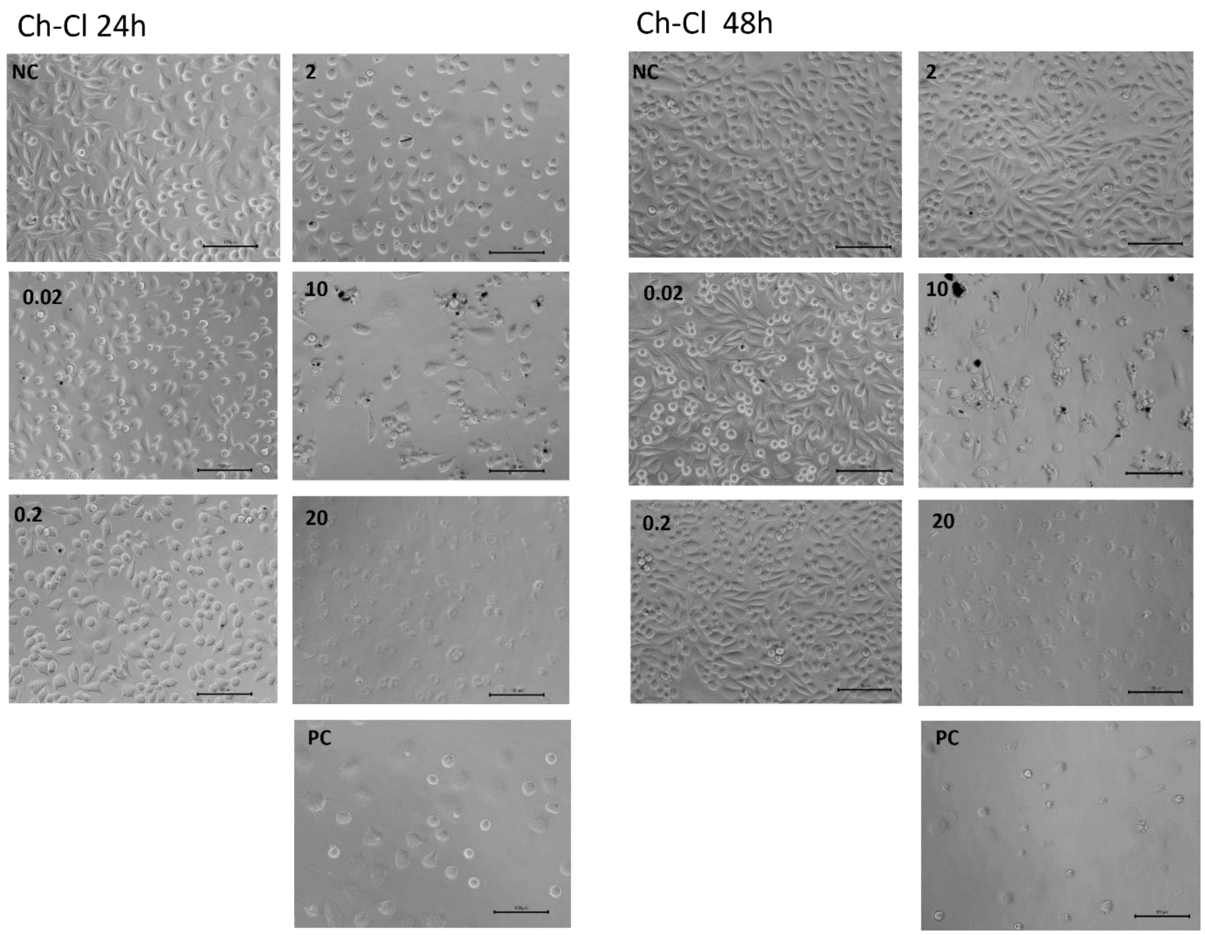

Figure S26: *Morphology of the L929 mouse fibroblasts cells after 24 h and 48 h exposure to Ch-Cl. NC negative control, PC positive control. The numbers indicate the concentration of Ch-Cl in mg/ml.*

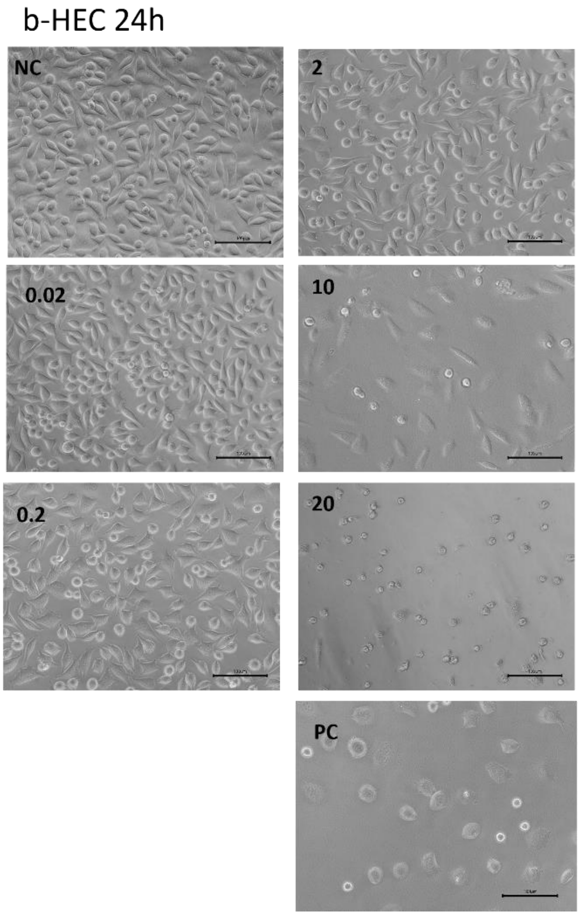

Figure S27: *Morphology of the L929 mouse fibroblasts cells after 24 h and 48 h exposure to b-HEC. NC negative control, PC positive control. The numbers indicate the concentration of b-HEC in mg/ml.*

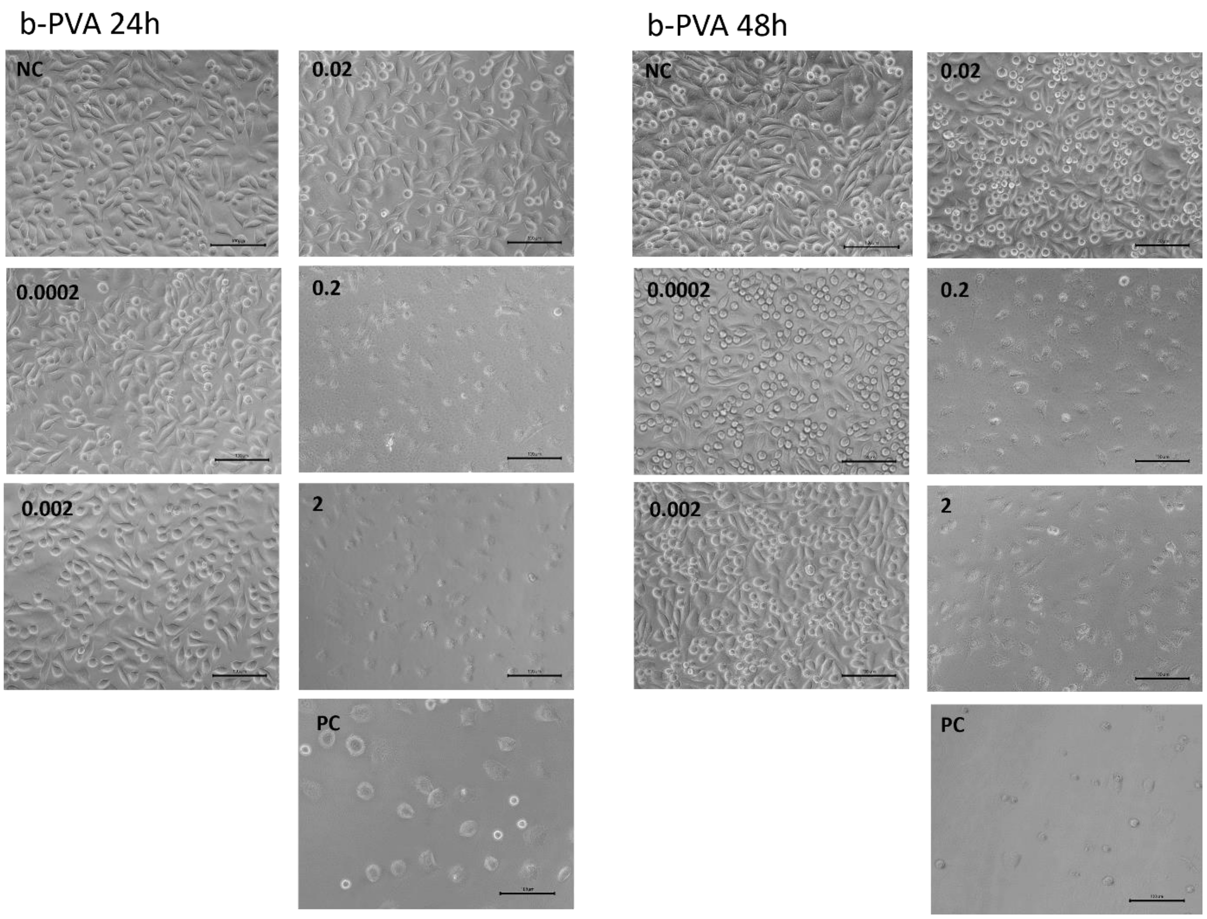

***Figure S28** Morphology of the L929 mouse fibroblasts cells after 24 h and 48 h exposure to b-PVA. NC negative control, PC positive control. The numbers indicate the concentration of b-PVA in mg/ml.*

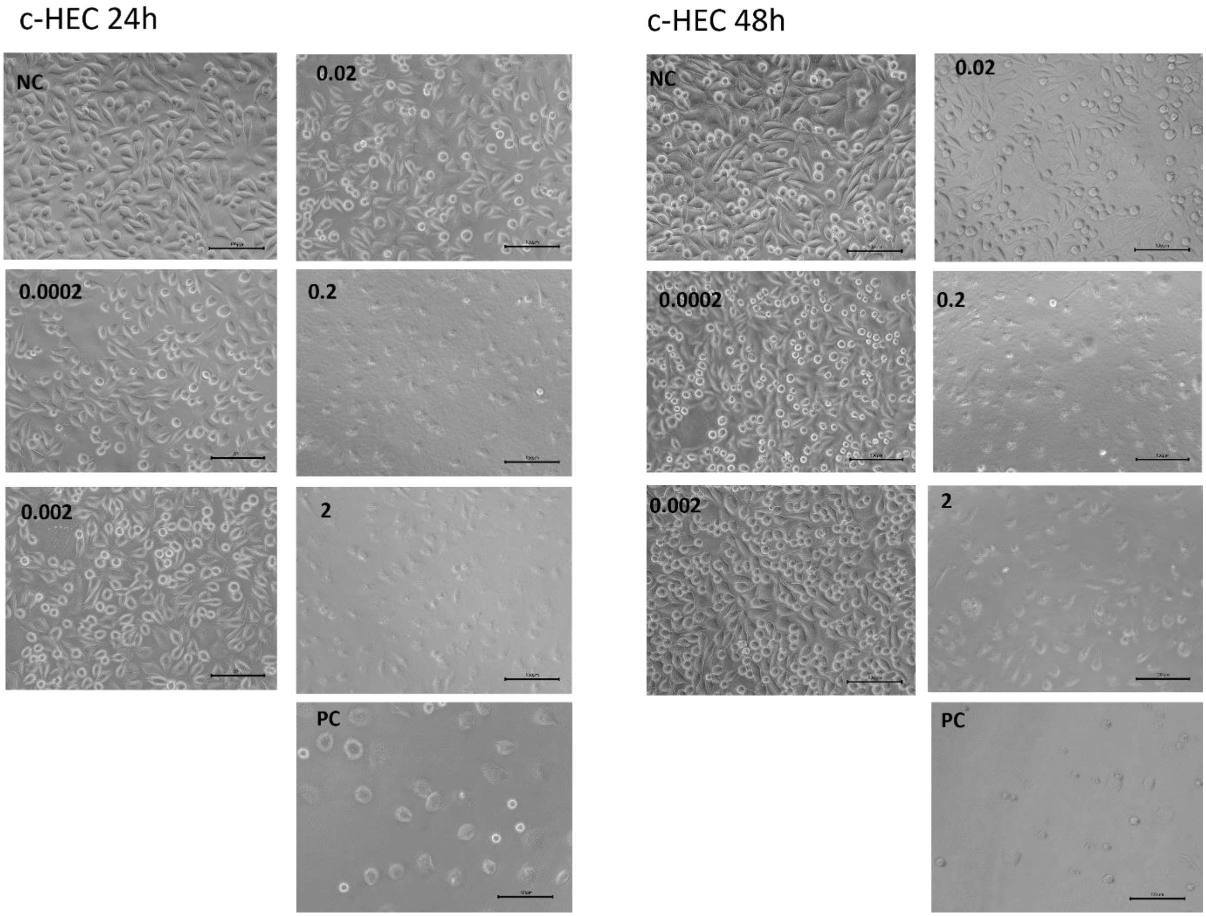

Figure S29: *Morphology of the L929 mouse fibroblasts cells after 24 h and 48 h exposure to c-HEC. NC negative control, PC positive control. The numbers indicate the concentration of c-HEC in mg/ml.*

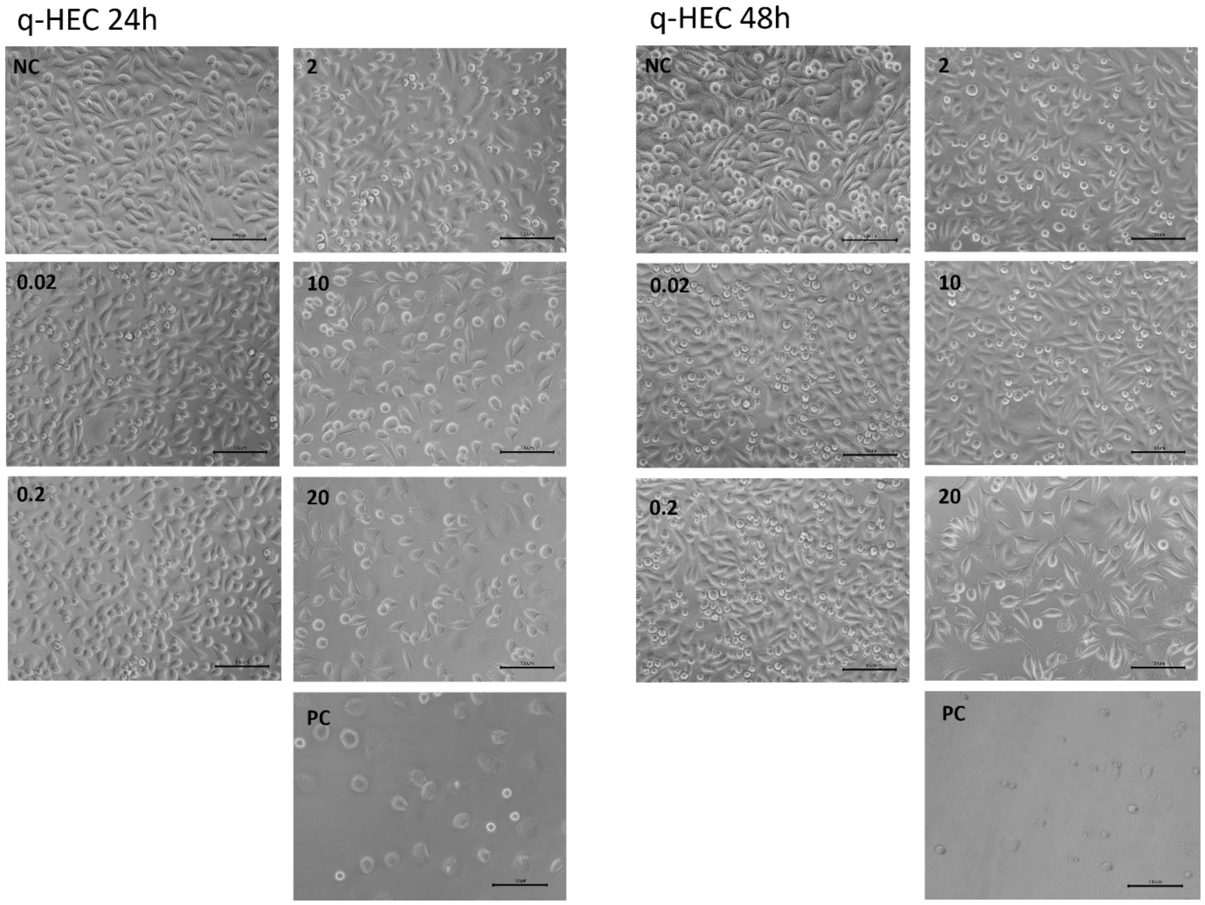

Figure S30: *Morphology of the L929 mouse fibroblasts cells after 24 h and 48 h exposure to q-HEC. NC negative control, PC positive control. The numbers indicate the concentration of q-HEC in mg/ml.*

Table S1: Antimicrobial properties of HEC, BET HCl, Ch-Cl. b-HEC, b-PVA, q-HEC, c-HEC against Gram positive Staphylococcus Aureus. Symbols depict: (-) complete growth inhibition, (+/-) complete to partial growth inhibition, (+) partial growth inhibition, (++) no growth inhibition, and (*) flocculation in the tested samples.

Staphylococcus Aureus												
		Concentration of substance in test tube (mg/mL)										
Sample		**20**	**10**	**5**	**2.5**	**1.25**	**0.62**	**0.31**	**0.16**	**0.08**	**0.04**	**0.02**
**BET HCl**		-	-	-	+	++	++	++	++	++	++	++
**Ch-Cl**		++	++	++	++	++	++	++	++	++	++	++
**b-HEC**		++	++	++	++	++	++	++	++	++	++	++
**b-PVA**	**Liquid media**	+/-	+/-	+/-	-	-	-	+/-	+/-	+	+	-
	**Solid media**	++	++	++	++	++	++	++	++	++	++	++
**q-HEC**				++	++	++	++	++	++	++	++	++
**c-HEC**	**Liquid media**		-	-	-	-	-	-	-	+/-*	+/-*	++
	**Solid media**						+	+	+			

Table S2 Antimicrobial properties of HEC, BET HCl, Ch-Cl. b-HEC, b-PVA, q-HEC, c-HEC against Gram negative Pseudomonas Aeruginosa. Symbols depict (-) complete growth inhibition, (+/-) complete to partial growth inhibition, (+) partial growth inhibition, (++) no growth inhibition, and (*) flocculation in the tested samples.

Pseudomonas Aeruginosa											
	Concentration of substance in test tube / (mg/mL)										
Sample	**20**	**10**	**5**	**2.5**	**1.25**	**0.62**	**0.31**	**0.16**	**0.08**	**0.04**	**0.02**
**BET HCl**	-	-	-	-	++	++	++	++	++	++	++
**Ch-Cl**	++	++	++	++	++	++	++	++	++	++	++
**b-HEC**	++	++	++	++	++	++	++	++	++	++	++
**b-PVA**	++	++	++	++	++	++	++	++	++	++	++
**q-HEC**			++	++	++	++	++	++	++	++	++
**c-HEC**		+*	+*	+*	+*	+*	+*	++*	++*	++	++
